# Kidney allograft rejection is associated with an imbalance of B cells, regulatory T cells and differentiated CD28-CD8+ T cells: analysis of a cohort of 1095 graft biopsies

**DOI:** 10.3389/fimmu.2023.1151127

**Published:** 2023-04-24

**Authors:** Hoa Le Mai, Nicolas Degauque, Marine Lorent, Marie Rimbert, Karine Renaudin, Richard Danger, Clarisse Kerleau, Gaelle Tilly, Anaïs Vivet, Sabine Le Bot, Florent Delbos, Alexandre Walencik, Magali Giral, Sophie Brouard

**Affiliations:** ^1^ Centre Hospitalier Universitaire (CHU) Nantes, Nantes Université, Institut National de la Santé et de la Recherche Médicale (INSERM), Center for Research in Transplantation and Translational Immunology, Unité mixte de recherche (UMR) 1064, Institut de Transplantation Urologie-Néphrologie (ITUN), Nantes, France; ^2^ Laboratoire d’Immunologie, Centre d’ImmunoMonitorage Nantes-Atlantique (CIMNA), CHU Nantes, Nantes, France; ^3^ Service d’Anatomie et Cytologie Pathologiques, CHU Nantes, Nantes, France; ^4^ Service de Néphrologie et Immunologie Clinique, CHU Nantes, Nantes, France; ^5^ Etablissement Français du Sang (EFS), Nantes, France; ^6^ Fondation Centaure (RTRS), Nantes, France

**Keywords:** kidney transplantation, rejection, B lymphocytes, Treg, CD28-CD8+ T cells

## Abstract

**Introduction:**

The human immune system contains cells with either effector/memory or regulatory functions. Besides the well-established CD4+CD25hiCD127lo regulatory T cells (Tregs), we and others have shown that B cells can also have regulatory functions since their frequency and number are increased in kidney graft tolerance and B cell depletion as induction therapy may lead to acute rejection. On the other hand, we have shown that CD28-CD8+ T cells represent a subpopulation with potent effector/memory functions. In the current study, we tested the hypothesis that kidney allograft rejection may be linked to an imbalance of effector/memory and regulatory immune cells.

**Methods:**

Based on a large cohort of more than 1000 kidney graft biopsies with concomitant peripheral blood lymphocyte phenotyping, we investigated the association between kidney graft rejection and the percentage and absolute number of circulating B cells, Tregs, as well as the ratio of B cells to CD28-CD8+ T cells and the ratio of CD28-CD8+ T cells to Tregs. Kidney graft biopsies were interpreted according to the Banff classification and divided into 5 biopsies groups: 1) normal/subnormal, 2) interstitial fibrosis and tubular atrophy grade 2/3 (IFTA), 3) antibody-mediated rejection (ABMR), 4) T cell mediated-rejection (TCMR), and 5) borderline rejection. We compared group 1 with the other groups as well as with a combined group 3, 4, and 5 (rejection of all types) using multivariable linear mixed models.

**Results and discussion:**

We found that compared to normal/subnormal biopsies, rejection of all types was marginally associated with a decrease in the percentage of circulating B cells (p=0.06) and significantly associated with an increase in the ratio of CD28-CD8+ T cells to Tregs (p=0.01). Moreover, ABMR, TCMR (p=0.007), and rejection of all types (p=0.0003) were significantly associated with a decrease in the ratio of B cells to CD28-CD8+ T cells compared to normal/subnormal biopsies. Taken together, our results show that kidney allograft rejection is associated with an imbalance between immune cells with effector/memory functions and those with regulatory properties.

## Introduction

In kidney transplantation, T cells and B cells participate in the alloimmune responses underlying the two main forms of graft rejection, T cell-mediated rejection (TCMR) and antibody-mediated rejection (ABMR), respectively. Although B cells are known for their effector functions, namely antibody production and antigen presentation, a randomized clinical trial in 2009 studying rituximab as B cell depleting-induction therapy was prematurely terminated because acute cellular rejection occurred in 6 of 8 treated kidney graft recipients, suggesting that B cells also have regulatory functions (1). In concordance with that observation, we (2–4) and others (5) independently reported that patients with drug-free kidney transplant tolerance have increased absolute number and relative frequency of circulating B lymphocytes compared to patients with stable graft function under immunosuppression, suggesting that B cells with regulatory functions may contribute to graft tolerance. Several studies have shown that human regulatory B cells (Bregs) are contained in the plasmablast (6) and transitional B cell (7) subsets defined as CD19+CD27+CD38+ and CD19+CD24hiCD38hi, respectively. IL-10 secretion has been shown to be an important mechanism of action of Bregs (IL-10+ Bregs) (8). Besides IL-10+ Bregs, we (9) and others (10) have identified another Breg subset that exerts their functions through the production of granzyme B (GZMB+ Bregs). Concordantly, kidney graft tolerance has been shown to be associated with an increase in circulating granzyme B-expressing B cells (9) and IL-10-expressing transitional B cells (5). In parallel, we (11, 12) and others (13) have reported that CD4 + CD25 +CD127loFoxP3+ Tregs were increased in tolerant patients compared to patients with stable renal function under immunosuppression. Therefore, it is likely that both Tregs and Bregs act in favor of immune tolerance in kidney transplantation (14, 15).

At the other end, among immune cells with effector/memory properties, CD28-CD8+ T cells are an intriguing T cell population. The interaction between CD28 on T cells and B7 on antigen-presenting cells provides the costimulatory signals necessary for T cell activation. However, chronic immune activation may down-regulate CD28 expression on CD8+ T cells. As a result, CD28-CD8+ T cells are increased in conditions or diseases associated with chronic antigenic exposure such as aging, autoimmune diseases, cancer, chronic infection, and transplantation (16, 17). Although some early studies in autoimmune disease models argued for a regulatory role of this T cell population (18, 19), we (20) and others (21) have demonstrated that human CD28-CD8+ T cells have strong cytotoxic effector function in response to alloantigen stimulation. Moreover, we also reported that CD28-CD8+ T cells are increased in kidney recipients with chronic rejection (20). To confirm those findings, we have established from 2008 to 2016 a large cohort of nearly 1500 kidney graft biopsies performed at Nantes University Hospital concomitant with a lymphocyte phenotyping by flow cytometry focusing on the CD28-CD8+ T cell population. By analyzing this cohort, we have shown that ABMR was associated with an increase in the percentage and absolute number of CD28-CD8+ T cells and those cells responded more vigorously to stimulations through T cell receptor (TCR) or FcγRIIIA (CD16) compared to their CD28+ counterparts, confirming their potent effector functions (22).

Having recognized the potential importance of B lymphocytes in transplant tolerance, from 2011 afterwards, we added B cells to the flow cytometry panel so that the later part of the aforementioned cohort contained 1095 kidney graft biopsies from 737 patients for whom the lymphocyte phenotyping included B cells in addition to CD28-CD8+ T cells and Tregs. In the current study, we analyzed this subcohort in order to explore whether there was an association between kidney graft rejection and the absolute number and relative frequency of B cells, Tregs, the ratio of B cells to CD28-CD8+ T cells, and the ratio of CD28-CD8+ T cells to Tregs. We found that kidney graft rejection was significantly associated with a decrease in the ratio of B cells to CD28-CD8+ T cells and an increase in the ratio of CD28-CD8+ T cells to Tregs. Taken together, our findings suggest that the imbalance between effector/memory and regulatory immune cells contributes to allograft rejection.

## Materials and methods

### Study design

As aforementioned, from 2008 to 2016, we established at Nantes University Hospital (Centre Hospitalier Universitaire or CHU de Nantes) a cohort of nearly 1500 kidney graft biopsies (protocol “Peribiopsy N° RC13_0251”) for which a peripheral lymphocyte phenotyping focusing on CD28-CD8+ T cells was performed at the time of biopsy (22). From 2011 afterward, we added B cells and Tregs to the panel so that the subcohort from 2011 to 2016 contained 1195 biopsies in which the frequency and absolute number of B cells, CD28-CD8+ T cells, and Tregs were available for analysis. Renal biopsies were interpreted by our renal pathologist (K.R.) based on the Banff 2017 Kidney Meeting Report (23) except for the diagnosis of chronic active TCMR which was based exclusively on the presence of chronic allograft arteriopathy as described in the Banff 2015 Kidney Meeting Report (24). More details on renal biopsy interpretation were described in our previous report (22). After having taken into account the clinical decision, the histological diagnoses were organized into 6 biopsy groups:

-Group 1 (n=802): normal/subnormal or interstitial fibrosis and tubular atrophy (IFTA) grade 1.
-Group 2 (n=56): IFTA grade 2 or 3.
-Group 3 (n=148): ABMR or borderline rejection treated as ABMR with plasma exchanges and intravenous immunoglobulins (IVIg), with or without rituximab.
-Group 4 (n=33): TCMR or borderline rejection treated as TMCR with corticosteroids.
-Group 5 (n=56): borderline rejection without treatment.
-Group 6 (n=100): other changes not considered to be caused by rejection.

Since our study focused on the association between lymphocyte phenotypes and rejection, 100 biopsies from group 6 (other changes) were excluded, leaving 1095 biopsies for the analysis, including 313 for cause biopsies and 414 and 368 three-month and 1-year surveillance biopsies, respectively. We investigated whether there was an association between the relative frequency and the absolute number of B cells, Tregs, the ratio of B cells to CD28-CD8+ T cells, and the ratio of CD28-CD8+ T cells to Tregs and the five biopsy groups. We also combined group 3 (ABMR), 4 (TCMR), and 5 (borderline rejection) into one group (hereinafter referred to as rejection of all types) and compared it with group 1 (normal/subnormal biopsy).

### Lymphocyte phenotyping of fresh blood

Each time a patient underwent a kidney graft biopsy at CHU de Nantes, a blood sample was drawn for laboratory analyses and an EDTA tube containing about 5 ml of blood was sent to the center for immunomonitoring (Centre d’Immunomonitorage Nantes-Atlantique or CIMNA) at CHU de Nantes for lymphocyte phenotyping. Flow cytometry was performed on fresh whole blood within 24 h after sampling and cells were analyzed on a BD FACS Canto II flow cytometer. To determine lymphocyte subset numbers, whole blood was stained in BD Trucount™ Tubes with the four-color monoclonal antibody reagents BD Multitest™ CD3/CD8/CD45/CD4 and BD Multitest™ CD3/CD19/CD16 + 56/CD45 according to the manufacturer’s instruction (BD Biosciences). To determine the percentage of Tregs and CD8+CD28- T cells, whole blood was incubated with the following fluorescence-conjugated monoclonal antibodies: anti-CD45-PerCP Cy5.5 (clone 2D1), anti-CD3-FITC (clone SK7), anti-CD4-APC (clone 13B8), anti-CD25-PE-Cy7 (clone 2A3), anti-CD127-PE (clone R34.34) anti-CD8-PE (clone B9.11), and anti-CD28-APC (clone CD28.2) (all from BD Biosciences except anti-CD4, anti-CD8, and anti-CD127 from Beckman Coulter) and then lyzed with FACS lyzing solution (BD Biosciences) (see **Supplementary Figure 1** for Treg gating strategy). B cells, Tregs, and CD28-CD8+ T cells were defined as CD3-CD19 +, CD3+CD4+CD25hiCD127lo, and CD3+CD8+CD28-, respectively and their percentages in total lymphocytes were reported. The absolute number of CD28-CD8+ T cells and Tregs were calculated by multiplying the absolute number of CD8+ T cells by the percentage of CD28-CD8+ T cells in CD8+ T cells and multiplying the absolute number of CD4+ T cells by the percentage of Tregs in CD4+ T cells, respectively. Absolute numbers of lymphocyte subsets were expressed as thousand per µl of blood.

### Phenotypic analysis of B cells using frozen PBMCs

Frozen PBMCs were thawed using CTL anti-aggregate buffer (Immunospot). 2x10^6^ PBMCs were stained with fixable viability stain 440UV (BD Biosciences), followed by fluorescence conjugated antibodies for cell surface markers including anti-CD3-BUV737 (clone UCHT1), anti-CD19-BV510 (clone HIB19), anti-CD24-BV711 (clone ML5), anti-CD38-BV785 (clone HIT2), anti-CD27-AF488 (clone QA17A18), anti-IgD-BV421 (clone IA6-2), anti-CD25-PE (clone BC96), and anti-CD9-PerCP Cy5.5 (clone H19a). Next, cells were permeabilized with Intracellular Fixation & Permeabilization Buffer Set (Thermo Fisher), stained with anti-granzyme B- PE Cy7 (clone QA18A28) (all antibodies from BioLegend except anti-CD3 from BD Biosciences), and acquired on a Celesta flow cytometer (BD Biosciences). Data were analyzed with Flowjo software version 10.8.0 (BD). The percentages of each B cell subset were compared among the 5 biopsy groups and between the normal/subnormal biopsy group and rejection of all types with the Kruskal-Wallis test and the Mann-Whitney test, respectively using the GraphPad Prism software version 5. All tests were 2-sided and p<0.05 was considered as statistical significant.

### Clinical data

Clinical data required for the analysis were extracted from the DIVAT (for “Données Informatisées et Validées en Transplantation”) database which was carried out prospectively, exhaustively, and independently by clinical research associates on key dates during post-operative follow-up of clinical and biological data of all incident transplanted patients at our institutes. The data are subject to an annual audit to warrant quality and completeness. Recipient characteristics include age, gender, transplantation rank (first transplantation or retransplantation), type of transplantation (renal or combined kidney/pancreas transplantation), year of transplantation, the initial kidney disease (possibly recurrent or not), the cytomegalovirus (CMV) serology, history of diabetes, history of arterial hypertension, history of cardiovascular disease, CMV serology, number of HLA-A-B-DR incompatibilities, ABO mismatch, donor-specific antibodies (DSA), and anti-HLA class I and II immunization. Donor characteristics include age, gender, and donor type (living or deceased). Baseline transplantation parameters were cold ischemia time, delayed graft function (DGF), induction therapy, and maintenance treatments [including cyclosporine A (CSA), tacrolimus, mammalian target of rapamycin (mTOR) inhibitors, mycophenolate mofetil (MMF), and corticosteroids]. Parameters collected at the time of biopsy were the reason for biopsy – for cause or surveillance biopsy (at 3 months or 1 year post-transplantation), serum creatinine, the rank of biopsy, post-transplantation time, histological diagnosis according to Banff 2017 classification and the whole details of the Banff elementary lesions, DSA, and type of treatment for each rejection episode, and the percentage and absolute number of total B cells, CD28-CD8+ T cells, and Tregs. The follow-up and the collection of data were stopped upon graft failure (defined as return to dialysis or retransplantation) or death.

### Statistical analyses using multivariable models

The characteristics at the time of biopsy between the five biopsy groups were described using median and interquartile range for continuous variables and frequency and proportion for categorical data. Six features were studied: the percentage and the absolute number of B cells and Tregs, the ratio between the absolute number of B cells and CD28-CD8+ T cells, and the ratio between the absolute number of CD28-CD8+ T cells and Tregs (only data from the lymphocyte phenotyping of fresh blood were included in the multivariate models). Since those features were non-Gaussian, we performed square root and logarithm transformation for each of those 6 features, plotted transformed data on histograms, and then selected the type of transformation that more closely resembled a normal distribution. In this way, square root transformation was used for the percentage and absolute number of B cells and Tregs whereas natural logarithm transformation was used for the ratio between the absolute number of B cells and CD28-CD8+ T cells and the ratio between the absolute number of CD28-CD8+ T cells and Tregs. We first performed linear mixed-effects models (random intercept per transplantation) to analyze the unadjusted effects of covariates on the studied feature (25). Covariates having p value less than 0.2 were then included into the multivariable models. We also forced into the multivariable models the following clinically important covariates (regardless of p value): recipient and donor age, recipient and donor sex, re-transplantation, recipient and donor CMV serology, cold ischemia time, time from transplantation to biopsy, reason for biopsy (for cause vs surveillance biopsy), induction therapy at transplantation, creatinine at biopsy, HLA-A, -B and-DR incompatibilities and anti-HLA class I and II immunization. We did not consider interaction. The residuals’ analyses were performed to check the models’ validities. In each model, we first tested if the outcome was significantly different in at least one of the biopsy groups using a likelihood ratio test. If significant, we explored which groups differed by performing the following comparisons: group 2 versus 1, group 3 versus 1, group 4 versus 1, and group 5 versus 1. We also compared rejection of all types (combination of group 1, 3, and 5) to group 1. Corrected p-values were determined using the Holm-Bonferroni method to control for the inflation of the type I error rate associated with multiple testing. Analyses were performed with R 4.0.3.

## Results

### Patient characteristics

Our analysis included 1095 kidney graft biopsies performed on 747 kidney or combined kidney/pancreas allograft from 737 patients. 73.2 percent (n=802) of biopsies were normal/subnormal (group 1), 5.1% (n=56) were grade 2/3 IFTA (group 2), 13.5% (n=148) were ABMR (group 3), 3% (n=33) were TCMR (group 4), and 5.1% (n=56) were untreated borderline rejection (group 5). The characteristics of the whole sample (1095 biopsies) as well as of each histological group from 1 to 5 were presented in **Table 1**.

**Table 1:** Characteristics of 1095 renal biopsies included in the analysis according their biopsy groups.

	Whole sample (n=1095)			Biopsy group 1 (n=802)			Biopsy group 2 (n=56)			Biopsy group 3 (n=148)			Biopsy group 4 (n=33)			Biopsy group 5 (n=56)		
	NA	n	%	NA	n	%	NA	n	%	NA	n	%	NA	n	%	NA	n	%
**Transplantation after 2008**	0	990	90.4	0	770	96.0	0	44	78.6	0	96	64.9	0	26	78.9	0	54	96.4
**Male recipient**	0	679	62.0	0	499	62.2	0	34	60.7	0	93	62.8	0	17	51.5	0	36	64.3
**Retransplantation**	0	191	17.4	0	139	17.3	0	7	12.5	0	38	25.7	0	3	9.1	0	4	7.1
**Renal transplantation**	0	992	90.6	0	724	90.3	0	53	94.6	0	139	93.9	0	27	81.8	0	49	87.5
**Recurrent initial disease**	0	248	22.7	0	179	22.3	0	11	19.6	0	41	27.7	0	3	9.1	0	14	25.0
**Delayed graft function**	13	345	31.9	8	240	30.2	2	23	42.6	3	46	31.7	0	14	42.4	0	22	39.3
**History of diabetes**	0	262	23.9	0	196	24.4	0	7	12.5	0	29	19.6	0	12	36.4	0	18	32.1
**History of hypertension**	0	961	87.8	0	706	88.0	0	48	85.7	0	128	86.5	0	30	90.9	0	49	87.5
**History of cardiovascular disease**	0	391	35.7	0	272	33.9	0	24	42.9	0	59	39.9	0	16	48.5	0	20	35.7
**Recipient/Donor CMV serology**	5			0			2			2			0			1		
**0**		379	34.8		284	35.4		12	22.2		53	36.3		13	39.4		17	30.9
**1**		224	20.6		155	19.3		20	37.0		30	20.6		7	21.2		12	21.8
** 2**		245	22.5		190	23.7		6	11.1		34	23.3		6	18.2		9	16.4
** 3**		242	22.2		173	21.6		16	29.6		29	19.9		7	21.2		17	30.9
**Male donor**	0	626	52.2	0	465	58.0	0	37	66.1	0	79	53.4	0	19	57.6	0	26	46.4
**Deceased donor**	0	967	88.3	0	704	87.8	0	52	92.9	0	130	87.8	0	29	87.9	0	52	92.9
**HLA-A-B-DR mismatches > 4**	0	255	23.3	0	187	23.3	0	10	17.9	0	36	24.3	0	10	30.3	0	12	21.4
**ABO mismatch**	0	22	2.0	0	18	2.2	0	0	0.0	0	3	2.0	0	0	0.0	0	1	1.8
**Depleting induction**	0	447	40.8	0	331	41.3	0	17	30.4	0	75	50.7	0	11	33.3	0	13	23.2
**Cyclosporine**	0	89	8.1	0	37	4.6	0	6	10.7	0	31	21.0	0	5	15.2	0	10	17.9
**Tacrolimus**	0	1004	91.7	0	764	95.3	0	50	89.3	0	116	78.4	0	28	84.9	0	46	82.1
**mTOR**	0	16	1.5	0	10	1.3	0	0	0.0	0	3	2.0	0	0	0.0	0	3	5.4
**Calcineurin inhibitors**	0	1090	99.5	0	799	99.6	0	56	100.0	0	147	99.3	0	33	100.0	0	55	98.2
**Corticosteroids**	0	948	86.6	0	696	86.8	0	45	80.4	0	130	87.8	0	26	78.8	0	51	91.1
**Positive anticlass I immunization**	1	339	31.0	0	247	30.8	1	13	23.6	0	66	44.6	0	6	18.2	0	7	12.5
**Positive anticlass II immunization**	1	310	28.3	0	219	27.3	1	9	16.4	0	65	43.9	0	8	24.2	0	9	16.1
**Positive DSA**	0	85	7.8	0	53	6.6	0	2	3.6	0	28	18.9	0	1	3.0	0	1	1.8
**Biopsy rank**	0			0			0			0			0			0		
** 1**		644	58.8		494	61.6		20	35.7		75	50.7		22	66.7		33	58.9
** 2**		372	34.0		274	34.2		29	51.8		42	28.4		7	21.2		20	35.7
** 3**		71	6.5		32	4.0		7	12.5		26	17.6		3	9.1		3	5.4
** 4**		7	0.6		2	0.3		0	0.0		4	2.7		1	3.0		0	0.0
** 5**		1	0.1		0	0.0		0	0.0		1	0.7		0	0.0		0	0.0
**For causes biopsy**	0	313	28.6	0	158	19.7	0	26	46.4	0	102	68.9	0	21	63.6	0	6	10.7
	NA	Median	Q1-Q3	NA	Median	Q1-Q3	NA	Median	Q1-Q3	NA	Median	Q1-Q3	NA	Median	Q1-Q3	NA	Median	Q1-Q3
**B cells (%)**	0	10.8	6.5-17.6	0	10.8	6.5-18.4	0	9.3	5.4-16.5	0	11.7	7.0-16.2	0	10.9	8.0-12.5	0	9.3	5.2-14.9
**B cells (absolute number)**	0	0.10	0.06-0.19	0	0.10	0.06-0.18	0	0.11	0.07-0.18	0	0.14	0.07-0.18	0	0.10	0.06-0.17	0	0.10	0.05-0.17
**Tregs (%)**	27	2.3	1.5-3.0	21	2.3	1.6-3.0	2	1.9	1.2-2.6	2	2.1	1.4-2.9	2	2.8	2.2-3.6	0	2.4	1.8-3.4
**Tregs (absolute number)**	28	0.02	0.01-0.04	21	0.02	0.01-0.04	2	0.02	0.01-0.03	2	0.02	0.01-0.04	2	0.03	0.02-0.04	0	0.03	0.02-0.04
**CD28-CD8+ T cells (%)**	11	6.6	3.0-15.4	8	5.9	2.7-13.4	1	12.7	4.6-20.9	2	9.4	3.6-23.8	0	7.7	3.6-16.8	0	7.6	3.2-16.2
**CD28-CD8+ T cells (absolute num)**	11	0.06	0.03-0.17	8	0.05	0.02-0.14	1	0.16	0.04-0.25	2	0.12	0.04-0.29	0	0.08	0.04-0.18	0	0.08	0.03-0.16
**Recipient age (years)**	0	51.0	40.0-63.0	0	51.0	41.0-63.0	0	53.5	38.8-63.3	0	50.0	34.0-61.0	0	52.0	36.0-63.0	0	52	25.0-64.0
**Cold ischemia time (hours)**	0	14.8	11.2-19.0	0	14.4	10.9-18.3	0	15.2	11.5-22.6	0	16.6	13.1-23.2	0	15.5	12.7-18.8	0	15.3	12.7-17.8
**Donor age (years)**	0	53.0	41.0-64.0	0	54.0	42.0-64.0	0	57	45.3-67.0	0	52.0	34.0-63.0	0	50.0	34.0-64.0	0	54.0	41.8-65.0
**Creatininemia at biopsy (µmol/l)**	12	139.0	110.0-179.0	9	133.0	106.0-169.0	2	175.0	141.3-247.0	0	163.5	132.8-214.5	0	199.0	141.0-291.0	1	125.0	105.0-160.0
**Post-transplantation time of the biopsy (years)**	0	0.97	0.26-1.03	0	0.36	0.26-1.01	0	1.03	1.00-5.21	0	1.87	0.99-7.00	0	0.55	0.25-2.29	0	0.28	0.26-1.01

CMV, Cytomegalovirus; Recipient/Donor CMV serology definition, 0, negative donor and recipient; 1, negative donor and positive recipient; 2, positive donor and negative recipient; 3, positive donor and recipient; DSA, donor-specific antibodies; HLA, human leukocyte antigens; Q, quartile; Q1, 25^th^ percentile; Q3, 75^th^ percentile; NA, not available (missing). %, percent of total lymphocytes; absolute number: thousand per µl. Biopsy group 1: normal/subnormal, group 2: interstitial fibrosis/tubular atrophy (IFTA) grade 2 or 3, group 3: ABMR, group 4, TCMR; group 5, borderline rejection.

### Rejection of all types is marginally associated with a decrease in the percentage of B cells in the peripheral blood

We first analyzed the unadjusted effects of covariates on the square root of B cell percentage using linear mixed models (**Supplementary Table 1**). Biopsy groups were not associated with the square root of B cell percentage (p=0.21) whereas compared to group 1, rejection of all types (combination of group 3, 4, and 5) was associated with a decrease in the square root of B cell percentage (p=0.04). The following covariates with p value of less than 0.2 were included into the multivariable model (in addition to clinically important covariates already forced into the model regardless of p value – see Materials and Methods): history of hypertension, ABO blood group mismatch, depleting induction, cyclosporine, tacrolimus, corticosteroids, and biopsy rank. We then performed multivariable linear mixed model analyses of the square root of B cell percentage and confirmed that biopsy groups were not associated with B cell percentage (p=0.22) (**Table 2**). Nevertheless, compared to group 1, rejection of all types was marginally associated with a decrease in the square root of B cell percentage, the adjusted mean difference was -0.18, 95% CI [-0.38, 0.01] (p=0.06) (**Table 3**). Next, we performed the same statistical analyses of the square root of B cell absolute number and found no association between biopsy groups and B cell absolute number (**Supplementary Table 2, 3**). The combined group (rejection of all types) also showed no difference in the square root of B cell absolute number compared to the normal/subnormal group, the adjusted mean difference was -0.01, 95% CI [-0.03, 0.02] (p=0.63) (**Supplementary Table 4**).

**Table 2:** Results of the multivariable linear mixed model of square root of B lymphocytes in percentage measured at the time of biopsy (5 biopsy groups).

	Adjusted mean difference	95% CI	p-value
**Biopsy group**			0.2224
**2 (vs. 1)**	-0.03	[-0.37; 0.31]	
**3 (vs. 1)**	-0.14	[-0.44; 0.11]	
**4 (vs. 1)**	-0.45	[-0.85; -0.05]	
**5 (vs. 1)**	-0.14	[-0.45; 0.18]	
**Recipient age (years)**	0.00	[-0.01; 0.01]	0.7448
**Male recipient**	-0.12	[-0.31; 0.06]	0.1979
**Retransplantation**	0.10	[-0.20; 0.40]	0.5070
**Cold ischemia time (hours)**	0.00	[-0.01; 0.01]	0.6806
**History of hypertension**	-0.20	[-0.48; 0.08]	0.1699
**Recipient/Donor CMV serology**			<0.0001
**1 (vs. 0)**	-0.45	[-0.69; -0.20]	
**2 (vs. 0)**	-0.16	[-0.40; 0.08]	
**3 (vs. 0)**	-0.33	[-0.57; -0.08]	
**Donor age (years)**	0.00	[-0.01; 0.01]	0.5639
**Male donor**	-0.09	[-0.27; 0.09]	0.3401
**HLA-A-B-DR mismatches > 4**	0.21	[-0.01; 0.42]	0.0610
**ABO mismatch**	-0.34	[-0.96; 0.28]	0.2781
**Depleting induction**	0.79	[0.56; 1.01]	<0.0001
**Cyclosporine**	-0.75	[-1.82; 0.32]	0.1710
**Tacrolimus**	0.04	[-1.04; 1.13]	0.9356
**Corticosteroids**	0.19	[-0.08; 0.46]	0.1687
**Positive anti-class I immunization**	-0.07	[-0.28; 0.14]	0.5199
**Positive anti-class II immunization**	-0.08	[-0.31; 0.15]	0.4923
**Biopsy rank**			0.1805
**2 (vs. 1)**	0.02	[-0.11; 0.16]	
**3 (vs. 1)**	-0.25	[-0.52; 0.02]	
**4 (vs. 1)**	-0.61	[-1.37; 0.14]	
**5 (vs. 1)**	-0.70	[-2.61; 1.20]	
**For causes biopsy**	0.17	[-0.02; 0.36]	0.0802
**Creatininemia at biopsy (μmol/l)**	0.00	[0.00; 0.00]	<0.0001
**Post-transplantation time of the biopsy (years)**	-0.01	[-0.04; 0.03]	0.6800

CI, confidence interval; CMV, cytomegalovirus; Recipient/Donor CMV serology definition. 0, negative donor and recipient; 1, negative donor and positive recipient; 2, positive donor and negative recipient; 3, positive donor and recipient; HLA ,human leucocyte antigens; Biopsy group 1, normal/subnormal; group 2, interstitial fibrosis/tubular atrophy (IFTA) grade 2 or 3; group 3, ABMR; group 4, TCMR; group 5, borderline rejection.

**Table 3:** Results of the multivariable linear mixed model of square root of B lymphocytes in percentage measured at the time of biopsy (Rejection of all types versus normal/subnormal).

	Adjusted mean difference	95% CI	p-value
**Rejection of all types vs. normal/subnormal**	-0.18	[-0.38; 0.01]	0.0623
**Recipient age (years)**	0.00	[-0.01; 0.01]	0.9417
**Male recipient**	-0.11	[-0.31; 0.08]	0.2409
**Retransplantation**	0.08	[-0.23; 0.38]	0.6220
**Cold ischemia time (hours)**	0.00	[-0.02; 0.01]	0.6222
**History of hypertension**	-0.16	[-0.45; 0.13]	0.2730
**Recipient/Donor CMV serology**			<0.0001
** 1 (vs. 0)**	-0.47	[-0.72; -0.22]	
** 2 (vs. 0)**	-0.16	[-0.40; 0.08]	
** 3 (vs. 0)**	-0.28	[-0.53; -0.03]	
**Donor age (years)**	0.00	[-0.01; 0.01]	0.7714
**Male donor**	-0.07	[-0.26; 0.12]	0.4562
**HLA-A-B-DR mismatches > 4**	0.21	[-0.01; 0.43]	0.0581
**ABO mismatch**	-0.34	[-0.96; 0.28]	0.2839
**Depleting induction**	0.81	[0.58; 1.04]	<0.0001
**Cyclosporine**	-0.73	[-1.81; 0.34]	0.1820
**Tacrolimus**	0.07	[-1.08; 1.10]	0.9893
**Corticosteroids**	0.10	[-0.10; 0.46]	0.1976
**Positive anti-class I immunization**	-0.08	[-0.30; 0.13]	0.4424
**Positive anti-class II immunization**	-0.10	[-0.34; 0.14]	0.4099
**Biopsy rank**			0.2436
** 2 (vs. 1)**	0.04	[-0.09; 0.17]	
** 3 (vs. 1)**	-0.21	[-0.49; 0.07]	
** 4 (vs. 1)**	-0.61	[-1.36; 0.14]	
** 5 (vs. 1)**	-0.57	[-2.47; 1.32]	
**For causes biopsy**	0.17	[-0.03; 0.37]	0.0886
**Creatininemia at biopsy (μmol/l)**	0.00	[0.00; 0.00]	<0.0001
**Post-transplantation time of the biopsy (years)**	-0.01	[-0.04; 0.02]	0.5666

CI, confidence interval; CMV, cytomegalovirus; Recipient/Donor CMV serology definition. 0, negative donor and recipient; 1, negative donor and positive recipient; 2, positive donor and negative recipient; 3, positive donor and recipient; HLA, human leucocyte antigens.

### B cell subset relative frequencies were not significantly different between biopsy groups

In order to perform an in-depth study into B cell subpopulations, we searched in our biocollection for cryopreserved PBMCs from the whole cohort and were able to retrieve a total of 334 frozen samples from 334 patients, including 279, 7, 23, 6, and 19 patients with normal/subnormal biopsy, IFTA grade 2 or 3, ABMR, TCMR, and borderline rejection, respectively. We determined the percentage of principal B cell subsets among total B cells (CD3-CD19+), including naïve (CD27-IgD+), switched memory (CD27+IgD-), non-switched memory (CD27+IgD+), non-conventional (CD27-IgD-), transitional B cells (CD24hiCD38hi), and plasmablasts (CD24loCD38hi), as well as of other B cells subsets shown to have regulatory properties such as CD25+ (26), CD9+ (27), and granzyme B+ (9, 10) B cells (see **Supplementary Figure 2** for the gating strategy). We first compared the percentages of each B cell subset among the 5 biopsy groups and found no statistically significant differences between those groups (data not shown). Next, we compared the B cell subset percentages between rejection of all types and normal/subnormal biopsies and did not find significant differences between the 2 groups. Only rejection of all types tended to be associated with an increase in switched memory B cells, but the difference did not reach statistical significance (p=0.08, Mann-Whitney test) (**Supplementary Table 5**).

### ABMR, TCMR, and rejection of all types are associated with a decrease in the ratio of B cells to CD28-CD8+ T cells

We recently demonstrated that ABMR was associated with an increase in the percentage and absolute number of the differentiated CD28-CD8+ T cells in the peripheral blood. We also showed that and CD28-CD8+ T cells contained higher percentage of effector/memory CD8+ T cells expressing CD45RA (TEMRA) defined as CCR7-CD45RA+ and had stronger effector functions compared to their CD28+ counterpart (22). In the current study, we asked whether the balance between B cells and CD28-CD8+ T cells had an impact on the occurrence of kidney graft rejection. For this purpose, we first analyzed the unadjusted effects of covariates on the natural logarithm of the ratio between the absolute number of peripheral B cells and CD28-CD8+ T cells (**Supplementary Table 6**). Next, we performed multivariable linear mixed model analyses and observed a significant association between the natural logarithm of the ratio between the absolute number of peripheral B cells and CD28-CD8+ T cells and biopsy groups (p=0.007) (**Table 4**). The adjusted mean difference between group 3 and group 1 and between group 4 and group 1 were -0.44, 95% CI [-0.69, -0.19] and -0.33, 95%CI [-0.73, -0.06], respectively. Similar results were obtained when we compared rejection of all types with normal/subnormal biopsy (group 1). The adjusted mean difference between rejection of all types and group 1 was -0.36, 95% CI [-0.55, -0.16] (p=0.0003) (**Table 5**). In other words, the ratio of B cells to CD28-CD8+ T cells was 43 percent higher in the normal/subnormal biopsy group compared to rejection of all types. Taken together, ABMR, TCMR, as well as rejection of all types were associated with a decrease in the ratio of B cells to CD28-CD8+ T cells measured in the peripheral blood at the time of biopsy compared normal/subnormal biopsies.

**Table 4:** Results of the multivariable linear mixed model of log of the ratio between B lymphocytes in absolute number and CD28-CD8+ T cells in absolute number measured at the time of biopsy (5 biopsy groups).

	Adjusted mean difference	95% CI	p-value
**Biopsy group**			0.0073
** 2 (vs. 1)**	-0.13	[-0.47; 0.21]	
** 3 (vs. 1)**	-0.44	[-0.69; -0.19]	
** 4 (vs. 1)**	-0.33	[-0.73; -0.06]	
** 5 (vs. 1)**	-0.21	[-0.51; 0.10]	
**Recipient age (years)**	0.00	[-0.01; 0.01]	0.9204
**Male recipient**	-0.03	[-0.22; 0.16]	0.7536
**Renal transplantation**	0.46	[0.10; 0.81]	0.0115
**Delayed graft function**	-0.09	[-0.29; 0.11]	0.3839
**Retransplantation**	-0.42	[-0.73; -0.11]	0.0072
**Cold ischemia time (hours)**	-0.02	[-0.03; 0.00]	0.0375
**Recipient/Donor CMV serology**			<0.0001
** 1 (vs. 0)**	-1.38	[-1.62; -1.14]	
** 2 (vs. 0)**	-0.69	[-0.93; -0.46]	
** 3 (vs. 0)**	-1.33	[-1.57; -1.09]	
**Donor age (years)**	-0.01	[-0.02; 0.00]	0.0098
**Male donor**	-0.13	[-0.32; 0.05]	0.1516
**Deceased donor**	0.13	[-0.25; 0.48]	0.5388
**HLA-A-B-DR mismatches > 4**	0.13	[-0.09; 0.34]	0.2511
**Depleting induction**	0.54	[0.31; 0.78]	<0.0001
**Cyclosporine**	-2.34	[-4.07; -0.60]	0.0085
**Tacrolimus**	-1.50	[-3.27; 0.28]	0.0980
**mTOR inhibitors**	0.44	[-0.35; 1.22]	0.2735
**Calcineurin inhibitors**	3.69	[1.42; 5.95]	0.0015
**Positive anti-class I immunization**	-0.04	[-0.25; 0.17]	0.7208
**Positive anti-class II immunization**	0.09	[-0.14; 0.32]	0.4491
**Biopsy rank**			<0.0001
** 2 (vs. 1)**	-0.30	[-0.43; -0.17]	
** 3 (vs. 1)**	-0.47	[-0.73; -0.21]	
** 4 (vs. 1)**	-1.16	[-1.89; -0.44]	
** 5 (vs. 1)**	-0.74	[-2.55; 1.08]	
**For causes biopsy**	0.13	[-0.06; 0.32]	0.1867
**Creatininemia at biopsy (100 μmol/l)**	0.08	[-0.01; 0.18]	0.0957
**Post-transplantation time of the biopsy (years)**	-0.01	[0.04; 0.03]	0.7013

CI, confidence interval; CMV, cytomegalovirus; HLA, human leucocyte antigens; mTOR, mammalian target of rapamycin; Recipient/Donor CMV serology definition. 0, negative donor and recipient; 1, negative donor and positive recipient; 2, positive donor and negative recipient; 3, positive donor and recipient; Log, natural logarithm; Biopsy group 1, normal/subnormal; group 2, interstitial fibrosis/tubular atrophy (IFTA) grade 2 or 3; group 3, ABMR; group 4, TCMR; group 5, borderline rejection.

**Table 5:** Results of the multivariable linear mixed model of log of the ratio between B lymphocytes in absolute number and CD28-CD8+ T cells in absolute number measured at the time of biopsy (Rejection of all types versus normal/subnormal).

	Adjusted mean difference	95% CI	p-value
**Rejection of all types vs. normal/subnormal**	-0.36	[-0.55; -0.16]	0.0003
**Recipient age (years)**	0.00	[-0.01; 0.01]	0.7902
**Male recipient**	-0.05	[-0.22; 0.15]	0.5821
**Renal transplantation**	0.44	[0.10; 0.81]	0.0162
**Delayed graft function**	-0.07	[-0.28; 0.12]	0.4845
**Retransplantation**	-0.42	[-0.72; -0.11]	0.0084
**Cold ischemia time (hours)**	-0.02	[-0.03; 0.00]	0.0499
**Recipient/Donor CMV serology**			<0.0001
** 1 (vs. 0)**	-1.37	[-1.61; -1.13]	
** 2 (vs. 0)**	-0.68	[-0.93; -0.46]	
** 3 (vs. 0)**	-1.28	[-1.56; -1.08]	
**Donor age (10 years)**	-0.01	[-0.02; 0.00]	0.0167
**Male donor**	-0.13	[-0.31; 0.06]	0.1636
**Deceased donor**	0.10	[-0.24; 0.49]	0.6097
**HLA-A-B-DR mismatches > 4**	0.12	[-0.10; 0.34]	0.2887
**Depleting induction**	0.56	[0.30; 0.77]	<0.0001
**Cyclosporine**	-2.35	[-4.09; -0.61]	0.0085
**Tacrolimus**	-1.52	[-3.27; 0.28]	0.0939
**mTOR inhibitors**	0.43	[-0.36; 1.20]	0.2867
**Calcineurin inhibitors**	3.63	[1.39; 5.92]	0.0018
**Positive anti-class I immunization**	-0.08	[-0.26; 0.16]	0.4445
**Positive anti-class II immunization**	0.07	[-0.15; 0.30]	0.5346
**Biopsy rank**			<0.0001
** 2 (vs. 1)**	-0.31	[-0.42; -0.16]	
** 3 (vs. 1)**	-0.50	[-0.74; -0.22]	
** 4 (vs. 1)**	-1.22	[-1.95; -0.51]	
** 5 (vs. 1)**	-0.83	[-2.65; 0.97]	
**For causes biopsy**	0.13	[-0.08; 0.29]	0.2085
**Creatininemia at biopsy (100 μmol/l)**	0.08	[-0.02; 0.18]	0.1030
**Post-transplantation time of the biopsy (years)**	-0.01	[0.04; 0.03]	0.5394

CI, confidence interval; CMV, cytomegalovirus; Recipient/Donor CMV serology definition. 0, negative donor and recipient; 1, negative donor and positive recipient; 2, positive donor and negative recipient; 3, positive donor and recipient. HLA, human leucocyte antigens; mTOR, mammalian target of rapamycin; Log, natural logarithm.

### Rejection of all types is associated with an increase in the ratio of CD28-CD8+ T cells to Tregs

Since we have previously shown that kidney tolerant patients had increased circulating Tregs (11, 12), in this study, we investigated the association between the 5 biopsy groups and Tregs, both in relative frequency and in absolute number. We first analyzed the unadjusted effects of covariates on the square root of Tregs in percentage and observed that biopsy groups were associated with the square root of Treg percentage (p=0.03) (**Supplementary Table 7**), however the association did not reach statistical significance when analyzed by multivariable linear mixed models (p=0.08) (**Supplementary Table 8**). We also found that biopsy groups were not associated with the square root of Treg absolute number (p=0.23 by multivariable analyses) (**Supplementary Tables 9, 10**). Similarly, multivariable analyses did not show statistically significant differences in Treg percentage and Treg absolute number between rejection of all types and normal/subnormal biopsy. The adjusted mean difference in the square root of Treg percentage and Treg absolute number between rejection of all types and normal/subnormal biopsy were 0.06, 95% CI [-0.02, 0.13], p=0.10 and 0.01, 95% CI [0.00, 0.02], p=0.089, respectively (**Supplementary Tables 11, 12**).

Next, we asked whether the balance between CD28-CD8+ T cells and Tregs was associated with kidney graft rejection. To this end, we first analyzed the unadjusted effects of covariates on the natural logarithm of the ratio between CD28-CD8+ T cells and Tregs and observed an association between this ratio and biopsy groups (p<0.0001) or rejection of all types (p=0.0002) (**Supplementary Table 13**). Next we performed multivariable linear mixed model analyses of the natural logarithm of the ratio between CD28-CD8+ T cells and Tregs and found a marginal association between this ratio and biopsy groups (p=0.06), especially the adjusted mean difference between group 3 and group 1 was 0.34, 95% CI [0.11, 0.58] (**Table 6**). More interestingly, multivariable analyses confirmed that rejection of all types was significantly associated with an increase in the ratio of CD28-CD8+ T cells to Tregs compared to normal/subnormal biopsy, the adjusted mean difference in the log of the ratio of CD28-CD8+ T cells/Tregs was 0.23, 95% CI [0.05, 0.41], p=0.01 (**Table 7**). In other words, the ratio of CD28-CD8+ T cells to Tregs was 26 percent higher in rejection of all types compared to the normal/subnormal biopsy group.

**Table 6:** Results of the multivariable linear mixed model of log of the ratio between CD28-CD8+ T cells in absolute number and Tregs in absolute number measured at the time of biopsy (5 biopsy groups).

	Adjusted mean difference	95% CI	p-value
**Biopsy group**			0.0673
** 2 (vs. 1)**	0.13	[-0.19; 0.44]	
** 3 (vs. 1)**	0.34	[0.11; 0.58]	
** 4 (vs. 1)**	0.09	[-0.29; 0.46]	
** 5 (vs. 1)**	0.10	[-0.19; 0.38]	
**Transplantation after 2008**	0.29	[-0.22; 0.79]	0.2695
**Recipient age (years)**	0.00	[-0.01; 0.01]	0.6200
**Male recipient**	-0.08	[-0.26; 0.09]	0.3576
**Retransplantation**	0.48	[0.19; 0.76]	0.0013
**Renal transplantation**	0.09	[-0.25; 0.42]	0.6064
**Recurrent initial disease**	0.07	[-0.13; 0.27]	0.5058
**Delayed graft function**	0.10	[-0.08; 0.29]	0.2799
**Cold ischemia time (hours)**	0.00	[-0.01; 0.02]	0.5476
**Recipient/Donor CMV serology**			<0.0001
** 1 (vs. 0)**	1.31	[1.08; 1.54]	
** 2 (vs. 0)**	0.67	[0.45; 0.89]	
** 3 (vs. 0)**	1.30	[1.07; 1.52]	
**Donor age (years)**	0.01	[0.00; 0.02]	0.0061
**Male donor**	0.13	[-0.04; 0.30]	0.1383
**Deceased donor**	0.19	[-0.15; 0.53]	0.2667
**HLA-A-B-DR mismatches > 4**	0.03	[-0.18; 0.23]	0.7951
**Depleting induction**	-0.03	[-0.26; 0.19]	0.7607
**Cyclosporine**	0.06	[-0.92; 1.04]	0.9023
**Tacrolimus**	-0.30	[-1.30; 0.69]	0.5518
**Corticosteroids**	0.19	[-0.06; 0.43]	0.1354
**Positive anti-class I immunization**	-0.05	[-0.25; 0.15]	0.6170
**Positive anti-class II immunization**	-0.13	[-0.34; 0.09]	0.2522
**Biopsy rank**			<0.0001
** 2 (vs. 1)**	0.50	[0.37; 0.62]	
** 3 (vs. 1)**	0.59	[0.35; 0.84]	
** 4 (vs. 1)**	0.73	[0.04; 1.42]	
** 5 (vs. 1)**	0.65	[-1.03; 2.34]	
**For causes biopsy**	0.14	[-0.04; 0.33]	0.1212
**Creatininemia at biopsy (100 μmol/l)**	0.00	[-0.10; 0.10]	0.9868
**Post-transplantation time of the biopsy (years)**	0.04	[-0.01; 0.09]	0.1101

CI, confidence interval; CMV, cytomegalovirus; HLA, human leucocyte antigens; Recipient/Donor CMV serology definition. 0, negative donor and recipient; 1, negative donor and positive recipient; 2, positive donor and negative recipient; 3, positive donor and recipient; Log, natural logarithm; Biopsy group 1, normal/subnormal; group 2, interstitial fibrosis/tubular atrophy (IFTA) grade 2 or 3; group 3, ABMR; group 4, TCMR; group 5, borderline rejection.

**Table 7:** Results of the multivariable linear mixed model of log of the ratio between CD28-CD8+ T cells in absolute number and Tregs in absolute number measured at the time of biopsy (Rejection of all types versus normal).

	Adjusted mean difference	95% CI	p-value
**Rejection of all types vs. normal/subnormal**	0.23	[0.05; 0.41]	0.0126
**Transplantation after 2008**	0.35	[-0.18; 0.88]	0.1976
**Recipient age (years)**	0.00	[-0.01; 0.01]	0.7945
**Male recipient**	-0.06	[-0.24; 0.12]	0.5383
**Retransplantation**	0.46	[0.16; 0.75]	0.0023
**Renal transplantation**	0.09	[-0.25; 0.43]	0.5986
**Recurrent initial disease**	0.08	[-0.12; 0.29]	0.3972
**Delayed graft function**	0.10	[-0.09; 0.29]	0.3008
**Cold ischemia time (hours)**	0.01	[-0.01; 0.02]	0.5023
**Recipient/Donor CMV serology**			<0.0001
** 1 (vs. 0)**	1.30	[1.07; 1.53]	
** 2 (vs. 0)**	0.64	[0.42; 0.87]	
** 3 (vs. 0)**	1.24	[1.01; 1.47]	
**Donor age (years)**	0.01	[0.00; 0.02]	0.0110
**Male donor**	0.13	[-0.04; 0.30]	0.1452
**Deceased donor**	0.18	[-0.17; 0.53]	0.3132
**HLA-A-B-DR mismatches > 4**	0.03	[-0.18; 0.23]	0.8057
**Depleting induction**	-0.03	[-0.26; 0.20]	0.7838
**Cyclosporine**	0.09	[-0.89; 1.06]	0.8641
**Tacrolimus**	-0.30	[-1.30; 0.69]	0.5489
**Corticosteroids**	0.15	[-0.11; 0.40]	0.2534
**Positive anti-class I immunization**	0.00	[-0.19; 0.20]	0.9683
**Positive anti-class II immunization**	-0.12	[-0.34; 0.10]	0.2796
**Biopsy rank**			<0.0001
** 2 (vs. 1)**	0.52	[0.39; 0.64]	
** 3 (vs. 1)**	0.65	[0.39; 0.91]	
** 4 (vs. 1)**	0.80	[0.10; 1.50]	
** 5 (vs. 1)**	0.80	[-0.91; 2.51]	
**For causes biopsy**	0.15	[-0.04; 0.34]	0.1192
**Creatininemia at biopsy (100 μmol/l)**	0.00	[-0.10; 0.11]	0.9830
**Post-transplantation time of the biopsy (years)**	0.04	[-0.01; 0.09]	0.0903

CI, confidence interval; CMV, cytomegalovirus; HLA, human leucocyte antigens; Recipient/Donor CMV serology definition. 0, negative donor and recipient; 1, negative donor and positive recipient; 2, positive donor and negative recipient; 3, positive donor and recipient; Log, natural logarithm.

Besides biopsy groups, as observed in the aforementioned tables (**Tables 2**–**7**), some other covariates, especially depleting induction and recipient/donor CMV serology, were also found to be statistically significant. Indeed, depleting induction has an impact on lymphocyte numbers and frequencies, especially when half of the biopsies were performed within the first post-transplantation year (**Table 1**). In this cohort, depleting induction therapy was mainly based on anti-thymocyte globulin (ATG), which profoundly depletes T cells but reduces other lymphocyte subsets in a much lesser extent. This may explain the finding that depleting induction was associated with a significant increase in B cell percentage (**Tables 2**, **3**) and in the ratio of B cells to CD28-CD8+ T cells (**Tables 4**, **5**). On the contrary, the ratio of CD28-CD8+ T cells to Tregs was not affected by depleting induction (**Tables 6**, **7**) since ATG depletes all T cell subpopulations.

Interestingly, the covariate recipient/donor CMV serology was also found to be statistically significant. In our multivariable linear mixed models, we compared transplant recipients having positive CMV serology (group 1: positive recipient/negative donor and group 3: positive recipient/positive donor) or having high risk to become CMV positive (group 2: negative recipient/positive donor) with the group of recipients having negative CMV serology and at low risk to become CMV positive (group 0: negative recipient/negative donor). We found that compared to the CMV group 0, the other groups had a significant decrease in B cell percentage (**Tables 2**, **3**), a significant decrease in the ratio of B cells to CD28-CD8+ T cells (**Tables 4**, **5**), and a significant increase in the ratio of CD28-CD8+ T cells to Tregs (**Tables 6**, **7**). Several recent studies have shown that kidney transplant patients with positive CMV serology have higher frequency of CD28-CD8+ T cells compared to those with negative CMV serology (28–30). Therefore, the decrease in the ratio of B cells to CD28-CD8+ T cells and the increase in the ratio of CD28-CD8+ T cells to Tregs can be explained at least in part by the increase in CD28-CD8+ T cells. On the other hand, the association between B cell frequency and CMV status is less well-documented (29, 31). The effect of CMV on lymphocyte phenotypes in transplant recipients is an interesting issue necessitating further research but is beyond the scope of this paper. In summary, the presence of covariates with statistical significance such as CMV serology and depleting induction does not affect our conclusion that certain blood lymphocyte phenotypes are significantly associated with kidney graft rejections because these covariates have been adjusted in the multivariable linear mixed models.

## Discussion

The association between peripheral blood lymphocyte phenotypes and kidney graft rejection has been a subject of many studies. However, most of the previous reports, including ours, were based on relatively small numbers of patients and univariate analyses. In the current study, we prospectively established a large cohort of 1095 kidney graft biopsies with concomitant lymphocyte phenotyping and data were analyzed using multivariable linear mixed models. We first compared each type of rejection to the normal/subnormal biopsy group. We next performed an additional analysis comparing the combined group, rejection of all types, to the normal/subnormal biopsy group to obtain additional results. The combined group with higher number of cases helps to increase statistical power. Moreover, from an immunological point of view, graft rejection usually requires the mobilization of different components of the immune system (32). For example, the principal mechanism of ABMR involves B cells and antibodies, but T cell help is indispensable for B cell differentiation and antibody production. It has been shown that kidney graft biopsies with ABMR also contain abundant T cells and a third of patients diagnosed with TCMR also have positive DSA (33).

First of all, our multivariable analysis showed a marginal association (p=0.06) between a decrease in the percentage of total B cell and rejection of all types (**Table 3**), in concordance with previous reports showing that preservation of the B-cell compartment favored kidney graft tolerance (2–5, 34). However, this finding is not conclusive and the changes in total B cells may not represent changes in Bregs. In order to perform a more detailed B cell phenotype analysis, we searched for cryopreserved PBMCs in our biocollection and were able to retrieve frozen samples in about one-third of the studied cohort. In kidney transplant patients, the most studied B cell subset with regulatory properties is CD4hiCD38hi transitional B cells. Several studies reported that patients with kidney graft rejections, especially ABMR, have a reduction in circulating transitional B cells compared to those with stable graft function (35–40). In this study, we performed univariate analyses of B cell phenotyping data from more than 300 patients and did not find any significant association between kidney graft rejections and the relative frequency of Breg subsets, including transitional, CD25+, CD9+, Granzyme B+ B cells, and plasmablasts. We found that rejection of all types tend to be associated with an increase in the percentage of CD27+IgD- switched memory B cells, but the difference was not statistically significant (p=0.08). Switched memory B cells have been reported to be increased in patients with chronic ABMR compared to stable kidney recipients (35). Future studies should provide a more comprehensive phenotyping of B cells with emphasize on Bregs. Since Bregs have been shown to exert their suppressive effects through different signaling pathways, including IL-10, IL-35, TGF-β, granzyme B, and PD-L1 [reviewed in (41–43)], there is no unique marker for Breg. Therefore, carefully designed B cell panels including many surface markers together with intracellular cytokine staining should better study the association between distinct Breg subsets and graft rejection.

We next investigated the relationship between peripheral blood CD4+CD25hiCD127lo Tregs and graft rejections. We and others previously reported that compared to patients with stable renal function, patients with acute rejection (44), chronic rejection (45, 46) or ABMR (40) had lower frequency and/or number of circulating Tregs. However, as aforementioned, those studies were based on small numbers of patients and univariate analyses. The current study based on multivariable analyses of more than 1000 graft biopsies did not show any significant association between circulating Treg relative frequency or absolute number and biopsy groups, either separately or combined (rejection of all types). Despite the important role of Tregs in modulating alloimmune responses in transplantation, attempts to investigate the potential of circulating Tregs as biomarkers in kidney transplantation have not been successful so far (47). Because CD4+CD25hiCD127lo Tregs are likely a heterogeneous population, detailed Treg phenotyping should provide more knowledge on the mechanisms of action of different Treg subsets in the transplant setting. For example, we have recently shown that memory Tregs defined as CD45RA-FoxP3hi have a central role in kidney transplant tolerance. Memory Tregs in tolerant patients have increased FoxP3 Treg-specific demethylated region (TSDR) demethylation and express high levels of the ectonucleotidase CD39 which can degrade adenosine triphosphate (ATP), a key factor in inflammation (11, 12). Therefore, lymphocyte phenotyping using additional markers to further define Treg subpopulations such as CD45RA, CD45RO, CD39, or methylation status (11, 12, 48, 49) might help to unveil a better correlation between circulating Treg subsets and graft rejections.

Since allograft rejections may occur when immune cells with effector/memory functions overbalance those with regulatory functions, we next investigate the association between the ratio of circulating B cells to CD28-CD8+ T cells as well as the ratio of CD28-CD8+ T cells to Tregs and kidney graft rejections. CD28-CD8+ T cells represent an important T cell population with effector/memory function since they are enriched in TEMRA and display potent cytotoxic properties (22, 50), whereas B cells have been shown to have regulatory function [reviewed in (14)]. Here we showed that ABMR, TMCR, and rejection of all types were significantly associated with a decrease in the ratio of B cells to CD28-CD8+ T cells. We also found that ABMR and rejection of all types were marginally and significantly associated with an increase in the ratio of CD28-CD8+ T cells to Tregs, respectively. Since we have previously shown that CD28-CD8+ T cells are significantly increased in ABMR and TCMR (22), it is likely that the increase in CD28-CD8+ T cells itself contributes to the increase in the ratio of CD28-CD8+ T cells to Tregs and the decrease in the ratio of B cells to CD28-CD8+ T cells in graft rejection. On the other hand, the differences in and B cells and Tregs between different biopsy groups did not reach statistical significance. Thus, the possible explanation for the changes in these two ratios is an increase in CD28-CD8+ T cells coupled with an absence of statistically significant changes in the other two populations. In other words, the increase in memory/effector T cells is not counterbalanced by a significant increase in other cell populations with potential regulatory properties. Taken together, the novel finding here is that graft rejection is associated not only with an increase in memory/effector T cells but also with an overbalance of memory/effector T cells over regulatory cells.

Relatively few studies have explored the impact of the balance between various circulating effector and regulatory cells on kidney graft outcome. For example, increased circulating follicular helper T cells (Tfh) to follicular regulatory T cells (Tfr) has been shown to be an independent risk factor for chronic allograft dysfunction (51). Another study found that a reduction in the ratio of IL-10-secreting (anti-inflammatory) to TNF-α-secreting (pro-inflammatory) transitional B cells was associated with subsequent deterioration of graft function (52). The results of our current study reemphasized the importance of the balance between effector/memory and regulatory immune cells in the maintenance of allograft acceptance.

Our study has some limitations. Based upon our publication in 2006, we selected only a few markers so that lymphocyte phenotyping on fresh blood could be carried out rapidly at our hospital laboratories at the same time as graft biopsy. Today, we know that more markers are necessary to define naïve/memory T cells, Treg, and Breg subsets. With the advent of modern flow cytometry allowing the label of up to 40 markers at the same time, carefully designed multicenter study using comprehensive antibody panels covering all Breg and Treg subsets should help to better study the association between kidney graft rejections and peripheral blood lymphocyte phenotypes. Finally, the exact relationship between circulating immune cell phenotypes and graft rejection is not clear. For example, the decrease of a subpopulation in the blood may result from either a real reduction or migration into the graft (53). Concomitant analysis of graft infiltrates may help to better understand the role of different leukocyte subpopulations in graft rejection.

Taken together, despite some limitations, our current study analyzing a large transplant biopsy cohort using multivariable linear mixed models sheds new insight into the underlying mechanisms of graft rejection, further supports the notion that rejection may be linked to the imbalance between effector/memory and regulatory immune cells, and paves the way for future research to discover new biomarkers for graft rejection and graft outcome.

## Data availability statement

The original contributions presented in the study are included in the article/**Supplementary Material**. Further inquiries can be directed to the corresponding authors.

## Ethics statement

The studies involving human participants were reviewed and approved by Commission nationale de l’informatique et des libertés (CNIL). The patients/participants provided their written informed consent to participate in this study.

## Author contributions

HM, ND, MR, KR, SB, and MG participate in the writing and revision of the manuscript. SB, MG, HM, ND, ML, and RD participate in the conceptualization and methodology. MR provides and verifies lymphocyte phenotyping data. KR interpretes kidney graft biopsies. FD and AW provide and verify patients’ laboratory data. ML perform statistical analyses. ND, GT, and AV design and perform detailed B cell phenotyping. CK perform data extraction from the DIVAT database. SL, RD, CK, HM, SB, and MG verify the extracted data. SB and MG acquire funding and supervise the study. All authors approved the final version of the manuscript. All authors contributed to the article and approved the submitted version.

## DIVAT Cohort Collaborators

(Medical Doctors, Surgeons, HLA Biologists) in Nantes: Gilles Blancho, Julien Branchereau, Diego Cantarovich, Agnès Chapelet, Jacques Dantal, Clément Deltombe, Lucile Figueres, Claire Garandeau, Magali Giral, Caroline Gourraud-Vercel, Maryvonne Hourmant, Georges Karam, Clarisse Kerleau, Christophe Masset, Delphine Kervela, Sabine Lebot, Aurélie Meurette, Simon Ville, Christine Kandell, Anne Moreau, Karine Renaudin, Anne Cesbron, Florent Delbos, Alexandre Walencik, and Anne Devis.

## References

[1] Clatworthy MR Watson CJ Plotnek G Bardsley V Chaudhry AN Bradley JA. B-cell-depleting induction therapy and acute cellular rejection. N Engl J Med (2009) 360(25):2683–5. doi: 10.1056/NEJMc0808481 PMC4143588 19535812

[2] Pallier A Hillion S Danger R Giral M Racapé M Degauque N. Patients with drug-free long-term graft function display increased numbers of peripheral b cells with a memory and inhibitory phenotype. Kidney Int (2010) 78(5):503–13. doi: 10.1038/ki.2010.162 20531452

[3] Sagoo P Perucha E Sawitzki B Tomiuk S Stephens DA Miqueu P. Development of a cross-platform biomarker signature to detect renal transplant tolerance in humans. J Clin Invest. (2010) 120(6):1848–61. doi: 10.1172/JCI39922 PMC2877932 20501943

[4] Louis S Braudeau C Giral M Dupont A Moizant F Robillard N. Contrasting CD25hiCD4+T cells/FOXP3 patterns in chronic rejection and operational drug-free tolerance. Transplantation (2006) 81(3):398–407. doi: 10.1097/01.tp.0000203166.44968.86 16477227

[5] Newell KA Asare A Kirk AD Gisler TD Bourcier K Suthanthiran M. Identification of a b cell signature associated with renal transplant tolerance in humans. J Clin Invest. (2010) 120(6):1836–47. doi: 10.1172/JCI39933 PMC2877933 20501946

[6] Matsumoto M Baba A Yokota T Nishikawa H Ohkawa Y Kayama H. Interleukin-10-producing plasmablasts exert regulatory function in autoimmune inflammation. Immunity (2014) 41(6):1040–51. doi: 10.1016/j.immuni.2014.10.016 25484301

[7] Blair PA Noreña LY Flores-Borja F Rawlings DJ Isenberg DA Ehrenstein MR. CD19(+)CD24(hi)CD38(hi) b cells exhibit regulatory capacity in healthy individuals but are functionally impaired in systemic lupus erythematosus patients. Immunity (2010) 32(1):129–40. doi: 10.1016/j.immuni.2009.11.009 20079667

[8] Fillatreau S Sweenie CH McGeachy MJ Gray D Anderton SM. B cells regulate autoimmunity by provision of IL-10. Nat Immunol (2002) 3(10):944–50. doi: 10.1038/ni833 12244307

[9] Chesneau M Michel L Dugast E Chenouard A Baron D Pallier A. Tolerant kidney transplant patients produce b cells with regulatory properties. J Am Soc Nephrol (2015) 26(10):2588–98. doi: 10.1681/ASN.2014040404 PMC4587683 25644114

[10] Lindner S Dahlke K Sontheimer K Hagn M Kaltenmeier C Barth TF. Interleukin 21-induced granzyme b-expressing b cells infiltrate tumors and regulate T cells. Cancer Res (2013) 73(8):2468–79. doi: 10.1158/0008-5472.CAN-12-3450 23384943

[11] Braza F Dugast E Panov I Paul C Vogt K Pallier A. Central role of CD45RA- Foxp3hi memory regulatory T cells in clinical kidney transplantation tolerance. J Am Soc Nephrol (2015) 26(8):1795–805. doi: 10.1681/ASN.2014050480 PMC4520169 25556168

[12] Durand M Dubois F Dejou C Durand E Danger R Chesneau M. Increased degradation of ATP is driven by memory regulatory T cells in kidney transplantation tolerance. Kidney Int (2018) 93(5):1154–64. doi: 10.1016/j.kint.2017.12.004 29455908

[13] Moraes-Vieira PM Silva HM Takenaka MC Monteiro SM Lemos F Saitovitch D. Differential monocyte STAT6 activation and CD4(+)CD25(+)Foxp3(+) T cells in kidney operational tolerance transplanted individuals. Hum Immunol (2010) 71(5):442–50. doi: 10.1016/j.humimm.2010.01.022 20122976

[14] Stolp J Turka LA Wood KJ. B cells with immune-regulating function in transplantation. Nat Rev Nephrology. (2014) 10(7):389–97. doi: 10.1038/nrneph.2014.80 24846332

[15] Braza F Durand M Degauque N Brouard S. Regulatory T cells in kidney transplantation: new directions? Am J Transplant. (2015) 15(9):2288–300. doi: 10.1111/ajt.13395 26234373

[16] Strioga M Pasukoniene V Characiejus D. CD8+ CD28- and CD8+ CD57+ T cells and their role in health and disease. Immunology (2011) 134(1):17–32. doi: 10.1111/j.1365-2567.2011.03470.x 21711350 PMC3173691

[17] Mou D Espinosa J Lo DJ Kirk AD. CD28 negative T cells: is their loss our gain? Am J Transplant. (2014) 14(11):2460–6. doi: 10.1111/ajt.12937 PMC4886707 25323029

[18] Najafian N Chitnis T Salama AD Zhu B Benou C Yuan X. Regulatory functions of CD8+CD28- T cells in an autoimmune disease model. J Clin Invest. (2003) 112(7):1037–48. doi: 10.1172/JCI17935 PMC198520 14523041

[19] Davila E Kang YM Park YW Sawai H He X Pryshchep S. Cell-based immunotherapy with suppressor CD8+ T cells in rheumatoid arthritis. J Immunol (2005) 174(11):7292–301. doi: 10.4049/jimmunol.174.11.7292 15905576

[20] Baeten D Louis S Braud C Braudeau C Ballet C Moizant F. Phenotypically and functionally distinct CD8+ lymphocyte populations in long-term drug-free tolerance and chronic rejection in human kidney graft recipients. J Am Soc Nephrol (2006) 17(1):294–304. doi: 10.1681/ASN.2005020178 16338967

[21] Lo DJ Weaver TA Stempora L Mehta AK Ford ML Larsen CP. Selective targeting of human alloresponsive CD8+ effector memory T cells based on CD2 expression. Am J Transplant. (2011) 11(1):22–33. doi: 10.1111/j.1600-6143.2010.03317.x 21070604 PMC3057516

[22] Mai HL Degauque N Le Bot S Rimbert M Renaudin K Danger R. Antibody-mediated allograft rejection is associated with an increase in peripheral differentiated CD28-CD8+ T cells - analyses of a cohort of 1032 kidney transplant recipients. EBioMedicine (2022) 83(104226). doi: 10.1016/j.ebiom.2022.104226 PMC9420477 35988467

[23] Haas M Loupy A Lefaucheur C Roufosse C Glotz D Seron D. The banff 2017 kidney meeting report: revised diagnostic criteria for chronic active T cell–mediated rejection, antibody-mediated rejection, and prospects for integrative endpoints for next-generation clinical trials. Am J Transplantation. (2018) 18(2):293–307. doi: 10.1111/ajt.14625 PMC5817248 29243394

[24] Loupy A Haas M Solez K Racusen L Glotz D Seron D. The banff 2015 kidney meeting report: current challenges in rejection classification and prospects for adopting molecular pathology. Am J Transplantation. (2017) 17(1):28–41. doi: 10.1111/ajt.14107 PMC5363228 27862883

[25] Laird NM Ware JH. Random-effects models for longitudinal data. Biometrics (1982) 38(4):963–74. doi: 10.2307/2529876 7168798

[26] Ibrahim EH Aly MG Opelz G Morath C Zeier M Süsal C. Higher CD19+CD25(+) bregs are independently associated with better graft function in renal transplant recipients. BMC Nephrol. (2021) 22(1):180. doi: 10.1186/s12882-021-02374-2 33993874 PMC8127305

[27] Brosseau C Durand M Colas L Durand E Foureau A Cheminant MA. CD9(+) regulatory b cells induce T cell apoptosis via IL-10 and are reduced in severe asthmatic patients. Front Immunol (2018) 9(3034). doi: 10.3389/fimmu.2018.03034 PMC6308143 30622536

[28] Daniel L Tassery M Lateur C Thierry A Herbelin A Gombert JM. Allotransplantation is associated with exacerbation of CD8 T-cell senescence: the particular place of the innate CD8 T-cell component. Front Immunol (2021) 12(674016). doi: 10.3389/fimmu.2021.674016 PMC8334557 34367138

[29] Wang L Rondaan C de Joode AAE Raveling-Eelsing E Bos NA Westra J. Changes in T and b cell subsets in end stage renal disease patients before and after kidney transplantation. Immun Aging (2021) 18(1):43. doi: 10.1186/s12979-021-00254-9 PMC8574047 34749733

[30] Pickering H Sen S Arakawa-Hoyt J Ishiyama K Sun Y Parmar R. NK and CD8+ T cell phenotypes predict onset and control of CMV viremia after kidney transplant. JCI Insight (2021) 6(21):e153175. doi: 10.1172/jci.insight.153175 34609965 PMC8663544

[31] Besançon-Watelet C De March AK Renoult E Kessler M Béné MC Faure GC. Early increase of peripheral b cell levels in kidney transplant recipients with CMV infection or reactivation. Transplantation (2000) 69(3):366–71. doi: 10.1097/00007890-200002150-00010 10706044

[32] Nankivell BJ Alexander SI. Rejection of the kidney allograft. N Engl J Med (2010) 363(15):1451–62. doi: 10.1056/NEJMra0902927 20925547

[33] Calvani J Terada M Lesaffre C Eloudzeri M Lamarthée B Burger C. *In situ* Multiplex immunofluorescence analysis of the inflammatory burden in kidney allograft rejection: a new tool to characterize the alloimmune response. Am J Transpl (2020) 20(4):942–53. doi: 10.1111/ajt.15699 31715060

[34] Silva HM Takenaka MC Moraes-Vieira PM Monteiro SM Hernandez MO Chaara W. Preserving the b-cell compartment favors operational tolerance in human renal transplantation. Mol Med (Cambridge Mass). (2012) 18(1):733–43. doi: 10.2119/molmed.2011.00281 PMC3409285 22252714

[35] Nouël A Ségalen I Jamin C Doucet L Caillard S Renaudineau Y. B cells display an abnormal distribution and an impaired suppressive function in patients with chronic antibody-mediated rejection. Kidney Int (2014) 85(3):590–9. doi: 10.1038/ki.2013.457 24284517

[36] Cherukuri A Rothstein DM Clark B Carter CR Davison A Hernandez-Fuentes M. Immunologic human renal allograft injury associates with an altered IL-10/TNF-α expression ratio in regulatory b cells. J Am Soc Nephrol (2014) 25(7):1575–85. doi: 10.1681/ASN.2013080837 PMC4073434 24610932

[37] Shabir S Girdlestone J Briggs D Kaul B Smith H Daga S. Transitional b lymphocytes are associated with protection from kidney allograft rejection: a prospective study. Am J Transplant. (2015) 15(5):1384–91. doi: 10.1111/ajt.13122 25808898

[38] Svachova V Sekerkova A Hruba P Tycova I Rodova M Cecrdlova E. Dynamic changes of b-cell compartments in kidney transplantation: lack of transitional b cells is associated with allograft rejection. Transpl Int (2016) 29(5):540–8. doi: 10.1111/tri.12751 26839984

[39] Salehi S Shahi A Afzali S Keshtkar AA Farashi Bonab S Soleymanian T. Transitional immature regulatory b cells and regulatory cytokines can discriminate chronic antibody-mediated rejection from stable graft function. Int immunopharmacology. (2020) 86(106750). doi: 10.1016/j.intimp.2020.106750 32652501

[40] Louis K Fadakar P Macedo C Yamada M Lucas M Gu X. Concomitant loss of regulatory T and b cells is a distinguishing immune feature of antibody-mediated rejection in kidney transplantation. Kidney Int (2022) 101(5):1003–16. doi: 10.1016/j.kint.2021.12.027 PMC9038633 35090879

[41] Long W Zhang H Yuan W Lan G Lin Z Peng L. The role of regulatory b cells in kidney diseases. Front Immunol (2021) 12(683926). doi: 10.3389/fimmu.2021.683926 PMC8183681 34108975

[42] Catalán D Mansilla MA Ferrier A Soto L Oleinika K Aguillón JC. Immunosuppressive mechanisms of regulatory b cells. Front Immunol (2021) 12(611795). doi: 10.3389/fimmu.2021.683926 PMC8118522 33995344

[43] Jansen K Cevhertas L Ma S Satitsuksanoa P Akdis M van de Veen W. Regulatory b cells, a to z. Allergy (2021) 76(9):2699–715. doi: 10.1111/all.14763 33544905

[44] Mirzakhani M Shahbazi M Akbari R Oliaei F Asgharpour M Nikoueinejad H. Reduced CD4(+) CD25(++) CD45RA(-) Foxp3(hi) activated regulatory T cells and its association with acute rejection in patients with kidney transplantation. Transpl Immunol (2020) 60(101290). doi: 10.1016/j.trim.2020.101290 32240775

[45] Braudeau C Racape M Giral M Louis S Moreau A Berthelot L. Variation in numbers of CD4+CD25highFOXP3+ T cells with normal immuno-regulatory properties in long-term graft outcome. Transpl Int (2007) 20(10):845–55. doi: 10.1111/j.1432-2277.2007.00537.x 17854443

[46] Akl A Jones ND Rogers N Bakr MA Mostafa A El Shehawy el M. An investigation to assess the potential of CD25highCD4+ T cells to regulate responses to donor alloantigens in clinically stable renal transplant recipients. Transpl Int (2008) 21(1):65–73. doi: 10.1111/j.1432-2277.2007.00560.x 17887959

[47] López-Hoyos M San Segundo D Brunet M. Regulatory T cells as biomarkers for rejection and immunosuppression tailoring in solid organ transplantation. Ther Drug monitoring. (2016) 38(Suppl 1):S36–42. doi: 10.1097/FTD.0000000000000265 26977998

[48] San Segundo D Millán O Muñoz-Cacho P Boix F Paz-Artal E Talayero P. High proportion of pretransplantation activated regulatory T cells (CD4+CD25highCD62L+CD45RO+) predicts acute rejection in kidney transplantation: results of a multicenter study. Transplantation (2014) 98(11):1213–8. doi: 10.1097/TP.0000000000000202 25083613

[49] McRae JL Chia JS Pommey SA Dwyer KM. Evaluation of CD4(+) CD25(+/-) CD39(+) T-cell populations in peripheral blood of patients following kidney transplantation and during acute allograft rejection. Nephrol (Carlton Vic). (2017) 22(7):505–12. doi: 10.1111/nep.12894 27517975

[50] Jacquemont L Tilly G Yap M Doan-Ngoc TM Danger R. Terminally differentiated effector memory CD8(+) T cells identify kidney transplant recipients at high risk of graft failure. J Am Soc Nephrol (2020) 31(4):876–91. doi: 10.1681/ASN.2019080847 PMC7191929 32165419

[51] Yan L Li Y Li Y Wu X Wang X Wang L. Increased circulating tfh to tfr ratio in chronic renal allograft dysfunction: a pilot study. BMC Immunol (2019) 20(1):26. doi: 10.1186/s12865-019-0308-x 31382877 PMC6683539

[52] Cherukuri A Salama AD Carter CR Landsittel D Arumugakani G Clark B. Reduced human transitional b cell T1/T2 ratio is associated with subsequent deterioration in renal allograft function. Kidney Int (2017) 91(1):183–95. doi: 10.1016/j.kint.2016.08.028 28029430

[53] Heidt S Vergunst M Anholts JDH Swings G Gielis EMJ Groeneweg KE. Presence of intragraft b cells during acute renal allograft rejection is accompanied by changes in peripheral blood b cell subsets. Clin Exp Immunol (2019) 196(3):403–14. doi: 10.1111/cei.13269 PMC6514375 30712266

