# Supplementary material for: Kidney allograft rejection is associated with an imbalance of B cells, regulatory T cells and differentiated CD28-CD8+ T cells: analysis of a cohort of 1095 graft biopsies
Source: Front Immunol. 2023 Apr 24;14:1151127. doi: 10.3389/fimmu.2023.1151127 (PMC10164960; doi:10.3389/fimmu.2023.1151127)

Supplementary Figure 1. Gating strategy for regulatory T cells (Treg). Whole blood was incubated with an antibody mix containing fluorescence-conjugated anti-CD45, anti-CD3, anti-CD4, anti-CD25, and anti-CD127 and then lyzed with BD FACS lyzing solution. Data were acquired with BD Canto II flow cytometer and analyzed using FlowJo software. Shown are representative FACS plots from a blood sample. Cells were gated as followed: singlets → lymphocytes (CD45 high SSC low) → CD3+CD4+ → CD25 high CD127 low. Numbers indicate percentage of the parent population.

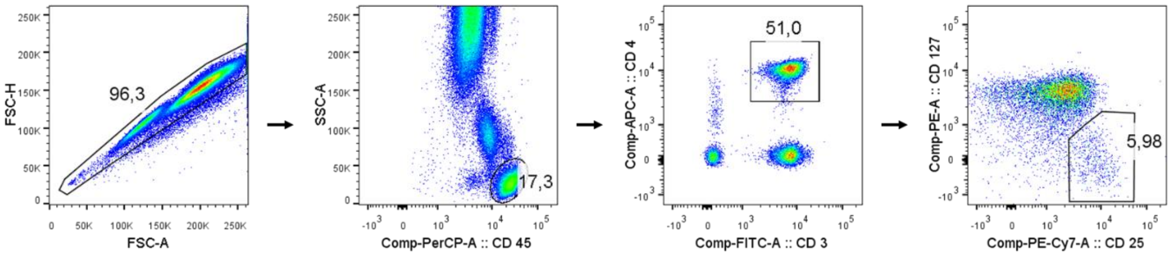

Supplementary Figure 2. B cell phenotypic analysis – Gating strategy. Cryopreserved PBMCs were thawed and stained with a fixable viability dye, followed by fluorescence-conjugated monoclonal antibodies directed against the cell surface markers CD3, CD9, CD19, CD24, CD25, CD27, CD38, and IgD. Cells were then permeabilized and stained for intracellular granzyme B (GZMb). Data were acquired with BD Celesta flow cytometer and analyzed using FlowJo software. Shown are representative FACS plots from a PBMC sample. Cells were gated as followed: lymphocytes → singlets → viable cells → CD3-CD19+ (B cells). B cells were then subdivided into several subpopulations: CD9+, CD25+, granzyme B+, CD27-IgD+ (naïve), CD27+IgD- (switched memory), CD27+IgD+ (non-switched memory), CD27-IgD- (non-conventional), CD24hiCD38hi (transitional), and CD24loCD38hi (plasmaplast).

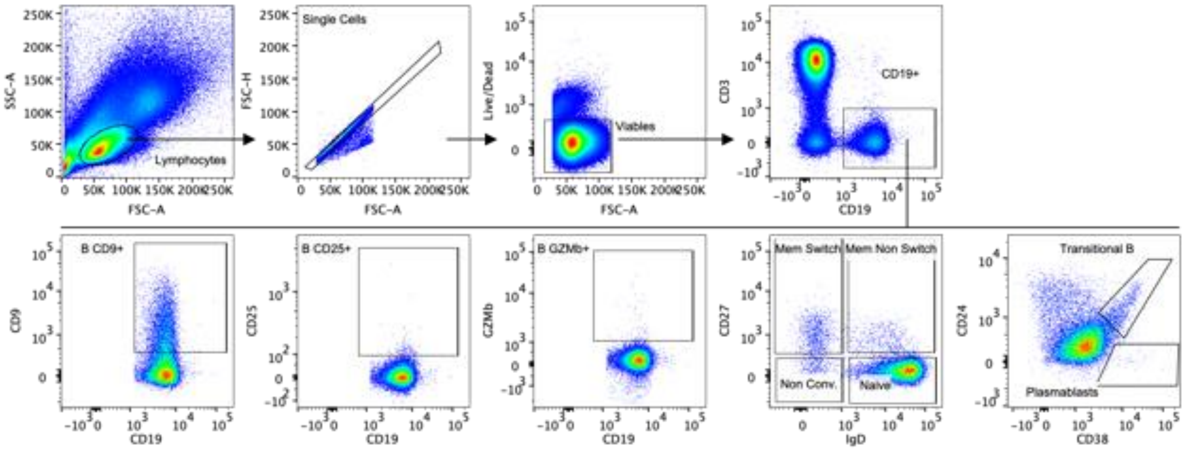

Supplement: Supplementary file 1 [file DataSheet_1.pdf]
