# Supplementary material for: Kidney allograft rejection is associated with an imbalance of B cells, regulatory T cells and differentiated CD28-CD8+ T cells: analysis of a cohort of 1095 graft biopsies
Source: Front Immunol. 2023 Apr 24;14:1151127. doi: 10.3389/fimmu.2023.1151127 (PMC10164960; doi:10.3389/fimmu.2023.1151127)
Supplement: Supplementary file 2 [file Table_1.docx]

**Supplementary Table 1**. Unadjusted effects of baseline covariates on square root of B lymphocytes in percentage measured at the time of biopsy, estimated from linear mixed models including random intercept.

|  | **Mean  difference** | **95% CI** | **p-value** |
| --- | --- | --- | --- |
| **Biopsy group** |  |  | 0.2194 |
| **2 (vs. 1)** | -0.14 | [-0.47 ; 0.20] |  |
| **3 (vs. 1)** | -0.10 | [-0.33 ; 0.13] |  |
| **4 (vs. 1)** | -0.28 | [-0.69 ; 0.12] |  |
| **5 (vs. 1)** | -0.32 | [-0.64 ; 0.01] |  |
| **Rejection of all types vs. normal/subnormal** | -0.19 | [-0.38 ; -0.01] | 0.0430 |
| **Transplantation after 2008** | 0.10 | [-0.20 ; 0.39] | 0.5250 |
| **Recipient age (years)** | 0.00 | [-0.01 ; 0.00] | 0.2220 |
| **Male recipient** | -0.17 | [-0.36 ; 0.02] | 0.0808 |
| **Retransplantation** | 0.58 | [0.34 ; 0.83] | <0.0001 |
| **Renal transplantation** | 0.07 | [-0.25 ; 0.38] | 0.6810 |
| **Recurrent initial disease** | 0.05 | [-0.18 ; 0.27] | 0.6930 |
| **Delayed graft function** | -0.07 | [-0.27 ; 0.13] | 0.4780 |
| **Cold ischemia time (hours)** | 0.00 | [-0.01 ; 0.01] | 0.8540 |
| **History of diabetes** | -0.05 | [-0.01 ; 0.01] | 0.6820 |
| **History of hypertension** | -0.22 | [-0.51 ; 0.07] | 0.1370 |
| **History of cardiovascular disease** | 0.12 | [-0.08 ; 0.31] | 0.2310 |
| **Recipient/Donor CMV serology** |  |  | 0.0237 |
| **1 (vs. 0)** | -0.35 | [-0.61 ; -0.10] |  |
| **2 (vs. 0)** | -0.18 | [-0.43 ; 0.07] |  |
| **3 (vs. 0)** | -0.31 | [-0.57 ; -0.06] |  |
| **Donor age (years)** | 0.00 | [-0.01 ; 0.00] | 0.2360 |
| **Male donos** | 0.03 | [-0.15 ; 0.22] | 0.7180 |
| **Deceased donor** | 0.13 | [-0.15 ; 0.42] | 0.3600 |
| **HLA-A-B-DR mismatches > 4** | 0.21 | [-0.02 ; 0.43] | 0.0681 |
| **ABO mismatch** | -0.43 | [-1.07 ; 0.20] | 0.1820 |
| **Depleting induction** | 0.82 | [0.64 ; 1.00] | <0.0001 |
| **Cyclosporine** | -0.69 | [-1.02 ; -0.36] | <0.0001 |
| **Tacrolimus** | 0.67 | [0.35 ; 1.00] | <0.0001 |
| **Corticosteroids** | 0.45 | [0.18 ; 0.72] | 0.0013 |
| **Positive anti-class I immunization** | 0.22 | [0.02 ; 0.42] | 0.0298 |
| **Positive anti-class II immunization** | 0.30 | [0.09 ; 0.51] | 0.0045 |
| **Positive DSA** | 0.00 | [-0.35 ; 0.35] | 0.9960 |
| **Biopsy rank** |  |  | 0.1150 |
| **2 (vs. 1)** | -0.01 | [-0.15 ; 0.12] |  |
| **3 (vs. 1)** | -0.31 | [-0.58 ; -0.05] |  |
| **4 (vs. 1)** | -0.64 | [-1.41 ; 0.13] |  |
| **5 (vs. 1)** | -0.25 | [-2.19 ; 1.70] |  |
| **For causes biopsy** | 0.17 | [0.00 ; 0.33] | 0.0433 |
| **Creatininemia at biopsy (μmol/l)** | 0.00 | [0.00 ; 0.00] | <0.0001 |
| **Post-transplantation time of the biopsy (years)** | -0.02 | [-0.04 ; 0.01] | 0.2050 |
| CI. confidence interval; CMV. cytomegalovirus; Recipient/Donor CMV serology definition. 0: negative donor and recipient; 1: negative donor and positive recipient; 2: positive donor and negative recipient; 3: positive donor and recipient. HLA. human leucocyte antigens. Biopsy group 1: normal/subnormal, group 2: interstitial fibrosis/tubular atrophy (IFTA) grade 2 or 3, group 3: ABMR, group 4: TCMR, group 5: borderline rejection. | | | |

**Supplementary Table 2**. Unadjusted effects of baseline covariates on square root of B lymphocytes in absolute number measured at the time of biopsy, estimated from linear mixed models including random intercept.

|  | **Mean  difference** | **95% CI** | **p-value** |
| --- | --- | --- | --- |
| **Biopsy group** |  |  | 0.0744 |
| **2 (vs. 1)** | 0.02 | [-0.02 ; 0.06] |  |
| **3 (vs. 1)** | 0.03 | [0.01 ; 0.06] |  |
| **4 (vs. 1)** | -0.02 | [-0.07 ; 0.03] |  |
| **5 (vs. 1)** | -0.02 | [-0.05 ; 0.02] |  |
| **Rejection of all types vs. normal/subnormal** | 0.01 | [-0.01 ; 0.03] | 0.3590 |
| **Transplantation after 2008** | -0.08 | [-0.11 ; 0.05] | <0.0001 |
| **Recipient age (years)** | 0.00 | [0.00 ; 0.00] | <0.0001 |
| **Male recipient** | 0.01 | [-0.01 ; 0.03] | 0.3270 |
| **Retransplantation** | -0.01 | [-0.03 ; 0.02] | 0.6520 |
| **Renal transplantation** | 0.06 | [-0.02 ; 0.14] | 0.2112 |
| **Recurrent initial disease** | 0.02 | [0.00 ; 0.05] | 0.0676 |
| **Delayed graft function** | 0.00 | [-0.03 ; 0.02] | 0.8060 |
| **Cold ischemia time (hours)** | 0.00 | [0.00 ; 0.00] | 0.9320 |
| **History of diabetes** | -0.03 | [-0.05 ; 0.00] | 0.0232 |
| **History of hypertension** | -0.02 | [-0.05 ; 0.01] | 0.2040 |
| **History of cardiovascular disease** | -0.01 | [-0.03 ; 0.01] | 0.4030 |
| **Recipient/Donor CMV serology** |  |  | 0.0775 |
| **1 (vs. 0)** | -0.04 | [-0.07 ; -0.01] |  |
| **2 (vs. 0)** | -0.02 | [-0.05 ; 0.00] |  |
| **3 (vs. 0)** | -0.03 | [-0.05 ; 0.00] |  |
| **Donor age (years)** | 0.00 | [0.00 ; 0.00] | 0.0012 |
| **Male donor** | 0.01 | [-0.02 ; 0.03] | 0.5810 |
| **Deceased donor** | -0.01 | [-0.04 ; 0.02] | 0.5470 |
| **HLA-A-B-DR mismatches > 4** | 0.01 | [-0.02 ; 0.03] | 0.5620 |
| **ABO mismatch** | -0.05 | [-0.12 ; 0.02] | 0.1730 |
| **Depleting induction** | -0.03 | [-0.05 ; -0.01] | 0.0062 |
| **Cyclosporine** | -0.01 | [-0.05 ; 0.03] | 0.6860 |
| **Tacrolimus** | 0.01 | [-0.03 ; 0.04] | 0.7440 |
| **Corticosteroids** | -0.02 | [-0.05 ; 0.01] | 0.2880 |
| **Positive anti-class I immunization** | -0.03 | [-0.05 ; 0.00] | 0.0172 |
| **Positive anti-class II immunization** | -0.02 | [-0.04 ; 0.01] | 0.1890 |
| **Positive DSA** | -0.06 | [-0.10 ; 0.02] | 0.0040 |
| **Biopsy rank** |  |  | <0.0001 |
| **2 (vs. 1)** | 0.04 | [0.03 ; 0.06] |  |
| **3 (vs. 1)** | 0.06 | [0.03 ; 0.09] |  |
| **4 (vs. 1)** | -0.02 | [-0.11 ; 0.07] |  |
| **5 (vs. 1)** | 0.05 | [-0.17 ; 0.27] |  |
| **For causes biopsy** | 0.03 | [0.01 ; 0.05] | 0.0005 |
| **Creatininemia at biopsy (μmol/l)** | 0.00 | [0.00 ; 0.00] | 0.4890 |
| **Post-transplantation time of the biopsy (years)** | 0.01 | [0.00 ; 0.01] | <0.0001 |
| CI. confidence interval; CMV. cytomegalovirus; Recipient/Donor CMV serology definition. 0: negative donor and recipient; 1: negative donor and positive recipient; 2: positive donor and negative recipient; 3: positive donor and recipient. HLA. human leucocyte antigens. DSA: donor-specific antibodies. Biopsy group 1: normal/subnormal, group 2: interstitial fibrosis/tubular atrophy (IFTA) grade 2 or 3, group 3: ABMR, group 4: TCMR, group 5: borderline rejection. | | | |

**Supplementary Table 3**. Results of the multivariable linear mixed model of square root of B lymphocytes in absolute number measured at the time of biopsy (5 biopsy groups).

|  | **Adjusted mean  difference** | **95% CI** | **p-value** |
| --- | --- | --- | --- |
| **Biopsy group** |  |  | 0.6416 |
| **2 (vs. 1)** | -0.01 | [-0.05 ; 0.03] |  |
| **3 (vs. 1)** | -0.01 | [-002 ; 0.04] |  |
| **4 (vs. 1)** | -0.03 | [-0.08 ; -0.02] | |
| **5 (vs. 1)** | -0.01 | [-0.05 ; 0.03] |  |
| **Recipient age (10 years)** | 0.00 | [-0.02 ; -0.01] | 0.0029 |
| **Male recipient** | 0.00 | [-0.02 ; 0.02] | 0.9883 |
| **Retransplantation** | 0.03 | [-0.01 ; 0.06] | 0.1459 |
| **Cold ischemia time (10 hours)** | 0.00 | [-0.02 ; 0.01] | 0.4060 |
| **History of diabetes** | -0.02 | [-0.04; 0.01] | 0.2298 |
| **Recipient/Donor CMV serology** |  |  | 0.2807 |
| **1 (vs. 0)** | -0.03 | [-0.06 ; 0.00] |  |
| **2 (vs. 0)** | -0.02 | [-0.04 ; 0.01] |  |
| **3 (vs. 0)** | -0.01 | [-0.04 ; 0.02] |  |
| **Donor age (10 years)** | 0.00 | [-0.01 ; 0.01] | 0.6721 |
| **Male donor** | 0.01 | [-0.02 ; 0.03] | 0.6229 |
| **HLA-A-B-DR mismatches > 4** | 0.01 | [-0.02 ; 0.03] | 0.5257 |
| **ABO mismatch** | -0.07 | [-0.15 ; 0.00] | 0.0590 |
| **Depleting induction** | -0.04 | [-0.07 ; -0.01] | 0.0072 |
| **Positive anti-class I immunization** | -0.01 | [-0.04 ; 0.01] | 0.3307 |
| **Positive anti-class II immunization** | 0.00 | [-0.03 ; 0.03] | 0.8416 |
| **Positive DSA** | -0.05 | [-0.09 ; 0.01] | 0.0275 |
| **Biopsy rank** |  |  | <0.0001 |
| **2 (vs. 1)** | 0.04 | [0.03 ; 0.06] |  |
| **3 (vs. 1)** | 0.04 | [0.01 ; 0.08] |  |
| **4 (vs. 1)** | -0.04 | [-0.12 ; 0.05] |  |
| **5 (vs. 1)** | 0.01 | [-0.21 ; 0.23] |  |
| **For causes biopsy** | 0.01 | [-0.01 ; 0.04] | 0.2283 |
| **Creatininemia at biopsy (100 μmol/l)** | 0.00 | [-0.01 ; 0.01] | 0.7293 |
| **Post-transplantation time of the biopsy (years)** | 0.00 | [0.00 ; 0.01] | 0.0137 |
| CI. confidence interval; CMV. cytomegalovirus; DSA. donor-specific antibodies; HLA. human leucocyte antigens. Recipient/Donor CMV serology definition. 0: negative donor and recipient; 1: negative donor and positive recipient; 2: positive donor and negative recipient; 3: positive donor and recipient. Biopsy group 1: normal/subnormal, group 2: interstitial fibrosis/tubular atrophy (IFTA) grade 2 or 3, group 3: ABMR, group 4: TCMR, group 5: borderline rejection. | | | |

**Supplementary Table 4**. Results of the multivariable linear mixed model of square root of B lymphocytes in absolute number measured at the time of biopsy (Rejection of all types versus normal/subnormal).

|  | **Adjusted mean  difference** | **95% CI** | **p-value** |
| --- | --- | --- | --- |
| **Rejection of all types vs. normal/subnormal** | -0.01 | [-0.03 ; 0.02] | 0.6309 |
| **Recipient age (10 years)** | -0.02 | [-0.03 ; -0.01] | 0.0023 |
| **Male recipient** | 0.00 | [-0.02 ; 0.02] | 0.8996 |
| **Retransplantation** | 0.03 | [-0.01 ; 0.06] | 0.1719 |
| **Cold ischemia time (10 hours)** | -0.01 | [-0.02 ; 0.01] | 0.2699 |
| **History of diabetes** | -0.01 | [-0.04; 0.01] | 0.2796 |
| **Recipient/Donor CMV serology** |  |  | 0.1377 |
| **1 (vs. 0)** | -0.03 | [-0.06 ; 0.00] |  |
| **2 (vs. 0)** | -0.02 | [-0.05 ; 0.01] |  |
| **3 (vs. 0)** | 0.00 | [-0.03 ; 0.03] |  |
| **Donor age (10 years)** | 0.00 | [-0.01 ; 0.01] | 0.6084 |
| **Male donor** | 0.01 | [-0.02 ; 0.03] | 0.5967 |
| **HLA-A-B-DR mismatches > 4** | 0.01 | [-0.02 ; 0.03] | 0.6250 |
| **ABO mismatch** | -0.07 | [-0.15 ; 0.00] | 0.0618 |
| **Depleting induction** | -0.04 | [-0.06 ; -0.01] | 0.0113 |
| **Positive anti-class I immunization** | -0.01 | [-0.04 ; 0.01] | 0.3057 |
| **Positive anti-class II immunization** | 0.00 | [-0.03 ; 0.03] | 0.8507 |
| **Positive DSA** | -0.04 | [-0.09 ; 0.00] | 0.0466 |
| **Biopsy rank** |  |  | <0.0001 |
| **2 (vs. 1)** | 0.04 | [0.03 ; 0.06] |  |
| **3 (vs. 1)** | 0.05 | [0.02 ; 0.08] |  |
| **4 (vs. 1)** | -0.03 | [-0.12 ; 0.06] |  |
| **5 (vs. 1)** | 0.02 | [-0.20 ; 0.24] |  |
| **For causes biopsy** | 0.01 | [-0.01 ; 0.04] | 0.2158 |
| **Creatininemia at biopsy (100 μmol/l)** | 0.00 | [-0.01 ; 0.01] | 0.6425 |
| **Post-transplantation time of the biopsy (years)** | 0.01 | [0.00 ; 0.01] | 0.0073 |
| CI. confidence interval; CMV. cytomegalovirus; Recipient/Donor CMV serology definition. 0: negative donor and recipient; 1: negative donor and positive recipient; 2: positive donor and negative recipient; 3: positive donor and recipient. HLA. human leucocyte antigens; DSA. donor-specific antibodies. | | | |

**Supplementary Table 5.** B cell phenotypic analysis – Comparison between rejection of all types and normal/subnormal biopsies.

| B cell subset  (% of total B cells) | Rejection of all types  (n = 48 ) | | Normal/subnormal  (n = 279 ) | | P value |
| --- | --- | --- | --- | --- | --- |
|  | Median | Q1 – Q3 | Median | Q1 – Q3 |  |
| Switched memory B cells | 13.3 | 7.89 – 24.15 | 11 | 5.98 – 19 | 0.08 |
| Naïve B cells | 71.9 | 49.1 – 83.6 | 73.3 | 60.1 – 85.2 | 0.15 |
| Non-switched memory B cells | 6.18 | 3.75 – 10.9 | 6.09 | 3.29 – 11.1 | 0.88 |
| Non-conventional B cells | 6.83 | 4.64 – 10.83 | 5.64 | 3.34 – 9.44 | 0.11 |
| Transitional B cells | 3.1 | 0.88 – 7.56 | 3.37 | 1.13 – 6.92 | 0.65 |
| Plasmablasts | 0.72 | 0.24 – 1.21 | 0.64 | 0.31 – 1.19 | 0.99 |
| CD25+ B cells | 0.4 | 0.25 – 0.75 | 0.41 | 0.23 – 0.8 | 0.96 |
| CD9+ B cells | 19.05 | 14.1 – 29.4 | 21.7 | 14.2 – 27.9 | 0.44 |
| Granzyme B+ B cells | 0.24 | 0.16 – 0.36 | 0.24 | 0.16 – 0.37 | 0.79 |

Q1. 25^th^ percentile; Q3. 75^th^ percentile; Mann-Whitney test.

**Supplementary Table 6**. Unadjusted effects of baseline covariates on log of the ratio between B lymphocytes and CD28-CD8+ T lymphocytes (both in absolute number) measured at the time of biopsy, estimated from linear mixed models including random intercept.

|  | **Mean  difference** | **95% CI** | **p-value** |
| --- | --- | --- | --- |
| **Biopsy group** |  |  | <0.0001 |
| **2 (vs. 1)** | -0.52 | [-0.86 ; -0.18] |  |
| **3 (vs. 1)** | -0.53 | [-0.79 ; -0.29] |  |
| **4 (vs. 1)** | -0.26 | [-0.67 ; 0.15] |  |
| **5 (vs. 1)** | -0.32 | [-0.64 ; 0.00] |  |
| **Rejection of all types vs. normal/subnormal** | -0.44 | [-0.63 ; -0.24] | <0.0001 |
| **Transplantation after 2008** | 0.08 | [-0.24 ; 0.40] | 0.6237 |
| **Recipient age (years)** | -0.01 | [-0.02 ; -0.01] | <0.0001 |
| **Male recipient** | -0.05 | [-0.26 ; 0.16] | 0.6160 |
| **Retransplantation** | -0.14 | [-0.40 ; 0.13] | 0.3090 |
| **Renal transplantation** | -0.24 | [-0.58 ; 0.10] | 0.1670 |
| **Recurrent initial disease** | -0.11 | [-0.35 ; 0.13] | 0.3820 |
| **Delayed graft function** | -0.28 | [-0.50 ; -0.06] | 0.0132 |
| **Cold ischemia time (hours)** | -0.03 | [-0.04 ; -0.01] | <0.0001 |
| **History of diabetes** | -0.09 | [-0.33 ; 0.15] | 0.4700 |
| **History of hypertension** | -0.08 | [-0.39 ; 0.23] | 0.6168 |
| **History of cardiovascular disease** | -0.02 | [-0.23 ; 0.19] | 0.8840 |
| **Recipient/Donor CMV serology** |  |  | <0.0001 |
| **1 (vs. 0)** | -1.37 | [-1.62 ; -1.12] |  |
| **2 (vs. 0)** | -0.75 | [-0.99 ; -0.50] |  |
| **3 (vs. 0)** | -1.37 | [-1.62 ; -1.12] |  |
| **Donor age (years)** | -0.01 | [-0.02 ; -0.01] | <0.0001 |
| **Male donor** | -0.03 | [-0.24 ; 0.17] | 0.7480 |
| **Deceased donor** | -0.31 | [-0.62 ; 0.01] | 0.0556 |
| **HLA-A-B-DR mismatches > 4** | 0.24 | [0.00 ; 0.48] | 0.0549 |
| **ABO mismatch** | 0.00 | [-0.69 ; 0.68] | 0.9897 |
| **Depleting induction** | 0.33 | [0.13 ; 0.54] | 0.0014 |
| **Cyclisporine** | -0.80 | [-1.15 ; -0.45] | <0.0001 |
| **Tacrolimus** | 0.78 | [0.43 ; 1.13] | <0.0001 |
| **Steroids** | 0.15 | [-0.14 ; 0.45] | 0.3137 |
| **Positive anti-class I immunization** | -0.05 | [-0.27 ; 0.00] | 0.6580 |
| **Positive anti-class II immunization** | -0.01 | [-0.23 ; 0.22] | 0.9520 |
| **Positive DSA** | 0.12 | [-0.28 ; 0.52] | 0.5460 |
| **Biopsy rank** |  |  | <0.0001 |
| **2 (vs. 1)** | -0.32 | [-0.44 ; -0.19] |  |
| **3 (vs. 1)** | -0.61 | [-0.86 ; -0.36] |  |
| **4 (vs. 1)** | -1.32 | [-2.03 ; -0.60] |  |
| **5 (vs. 1)** | -0.91 | [-2.71 ; 0.90] |  |
| **For causes biopsy** | -0.05 | [-0.22 ; 0.12] | 0.5740 |
| **Creatininemia at biopsy (μmol/l)** | 0.00 | [0.00 ; 0.00] | 0.9840 |
| **Post-transplantation time of the biopsy (years)** | -0.04 | [-0.07 ; -0.01] | 0.0028 |
| CI. confidence interval; CMV. cytomegalovirus; Recipient/Donor CMV serology definition. 0: negative donor and recipient; 1: negative donor and positive recipient; 2: positive donor and negative recipient; 3: positive donor and recipient. HLA. human leucocyte antigens. DSA: donor-specific antibodies. Biopsy group 1: normal/subnormal, group 2: interstitial fibrosis/tubular atrophy (IFTA) grade 2 or 3, group 3: ABMR, group 4: TCMR, group 5: borderline rejection. | | | |

**Supplementary Table 7**. Unadjusted effects of baseline covariates on square root of Tregs in percentage measured at the time of biopsy, estimated from linear mixed models including random intercept.

|  | **Mean  difference** | **95% CI** | **p-value** |
| --- | --- | --- | --- |
| **Biopsy group** |  |  | 0.0341 |
| **2 (vs. 1)** | -0.10 | [-0.22 ; 0.02] |  |
| **3 (vs. 1)** | -0.05 | [-0.13 ; 0.03] |  |
| **4 (vs. 1)** | 0.13 | [-0.02 ; 0.29] |  |
| **5 (vs. 1)** | 0.09 | [-0.02 ; 0.21] |  |
| **Rejection of all types vs. normal/subnormal** | 0.01 | [-0.05 ; 0.08] | 0.7250 |
| **Transplantation after 2008** | 0.06 | [-0.04 ; 0.16] | 0.2400 |
| **Recipient age (years)** | 0.00 | [0.00 ; 0.00] | 0.4150 |
| **Male recipient** | 0.01 | [-0.06 ; 0.07] | 0.9240 |
| **Retransplantation** | -0.12 | [-0.20 ; -0.04] | 0.0042 |
| **Renal transplantation** | -0.16 | [-0.26 ; -0.06] | 0.0019 |
| **Recurrent initial disease** | -0.03 | [-0.10 ; 0.04] | 0.4240 |
| **Delayed graft function** | -0.08 | [-0.14 ; -0.01] | 0.0246 |
| **Cold ischemia time (hours)** | 0.00 | [-0.01 ; 0.00] | 0.4310 |
| **History of diabetes** | 0.07 | [0.00 ; 0.14] | 0.0668 |
| **History of hypertension** | 0.04 | [-0.05 ; 0.13] | 0.4140 |
| **History of cardiovascular disease** | -0.02 | [-0.08 ; 0.05] | 0.5730 |
| **Recipient/Donor CMV serology** |  |  | 0.0047 |
| **1 (vs. 0)** | -0.11 | [-0.19 ; -0.02] |  |
| **2 (vs. 0)** | -0.12 | [-0.20 ; -0.03] |  |
| **3 (vs. 0)** | -0.13 | [-0.21 ; -0.05] |  |
| **Donor age (years)** | 0.00 | [0.00 ; 0.00] | 0.0347 |
| **Male donor** | -0.01 | [-0.07 ; 0.05] | 0.7990 |
| **Deceased donor** | -0.07 | [-0.16 ; 0.03] | 0.1480 |
| **HLA-A-B-DR mismatches > 4** | 0.03 | [-0.04 ; 0.10] | 0.4290 |
| **ABO mismatch** | 0.04 | [-0.18 ; 0.25] | 0.7310 |
| **Depleting induction** | -0.02 | [-0.09 ; 0.04] | 0.4520 |
| **Cyclosporine** | -0.07 | [-0.19 ; 0.34] | 0.2760 |
| **Tacrolimus** | 0.07 | [-0.05 ; 0.19] | 0.3000 |
| **Corticosteroids** | -0.08 | [-0.16 ; 0.01] | 0.0955 |
| **Positive anti-class I immunization** | -0.01 | [-0.08 ; 0.06] | 0.7800 |
| **Positive anti-class II immunization** | -0.06 | [-0.12 ; 0.01] | 0.1050 |
| **Positive DSA** | -0.01 | [-0.13 ; 0.10] | 0.8430 |
| **Biopsy rank** |  |  | 0.0176 |
| **2 (vs. 1)** | -0.06 | [-0.11 ; -0.01] |  |
| **3 (vs. 1)** | -0.14 | [-0.24 ; -0.04] |  |
| **4 (vs. 1)** | 0.07 | [-0.22 ; 0.35] |  |
| **5 (vs. 1)** | -0.29 | [-1.03 ; 0.44] |  |
| **For causes biopsy** | -0.15 | [-0.21 ; -0.09] | <0.0001 |
| **Creatininemia at biopsy (μmol/l)** | 0.00 | [0.00 ; 0.00] | <0.0001 |
| **Post-transplantation time of the biopsy (years)** | -0.01 | [-0.02 ; 0.00] | 0.0796 |
| CI. confidence interval; CMV. cytomegalovirus; Recipient/Donor CMV serology definition. 0: negative donor and recipient; 1: negative donor and positive recipient; 2: positive donor and negative recipient; 3: positive donor and recipient. HLA. human leucocyte antigens; DSA. donor-specific antibodies. Biopsy group 1: normal/subnormal, group 2: interstitial fibrosis/tubular atrophy (IFTA) grade 2 or 3, group 3: ABMR, group 4: TCMR, group 5: borderline rejection. | | | |

**Supplementary Table 8**. Results of the multivariable linear mixed model of square root of Tregs in percentage measured at the time of biopsy (5 biopsy groups).

|  | **Adjusted mean  difference** | **95% CI** | **p-value** |
| --- | --- | --- | --- |
| **Biopsy group** |  |  | 0.0804 |
| **2 (vs. 1)** | -0.02 | [-0.14 ; 0.10] |  |
| **3 (vs. 1)** | 0.02 | [-0.07 ; 0.11] |  |
| **4 (vs. 1)** | 0.20 | [0.05 ; 0.35] |  |
| **5 (vs. 1)** | 0.06 | [-0.05 ; 0.18] |  |
| **Recipient age (years)** | 0.00 | [0.00 ; 0.01] | 0.1154 |
| **Male recipient** | 0.03 | [-0.04 ; 0.09] | 0.4003 |
| **Retransplantation** | -0.08 | [-0.19 ; 0.03] | 0.1463 |
| **Renal transplantation** | -0.15 | [-0.29 ; 0.00] | 0.0483 |
| **Delayed graft function** | -0.04 | [-0.11 ; 0.03] | 0.2317 |
| **Cold ischemia time (hours)** | 0.01 | [0.00 ; 0.01] | 0.0320 |
| **History of diabetes** | 0.00 | [-0.09 ; 0.08] | 0.9303 |
| **Recipient/Donor CMV serology** |  |  | 0.0120 |
| **1 (vs. 0)** | -0.09 | [-0.18 ; -0.01] |  |
| **2 (vs. 0)** | -0.11 | [-0.19 ; -0.03] |  |
| **3 (vs. 0)** | -0.12 | [-0.20 ; -0.03] |  |
| **Donor age (years)** | 0.00 | [-0.01 ; 0.00] | 0.0588 |
| **Male donor** | -0.01 | [-0.08 ; 0.05] | 0.6658 |
| **Deceased donor** | -0.13 | [-0.26 ; -0.01] | 0.0411 |
| **HLA-A-B-DR mismatches > 4** | -0.02 | [-0.09 ; 0.06] | 0.6757 |
| **Depleting induction** | 0.00 | [-0.09 ; 0.08] | 0.9155 |
| **Corticosteroids** | -0.11 | [-0.20 ; -0.02] | 0.0191 |
| **Positive anti-class I immunization** | 0.03 | [-0.04 ; 0.11] | 0.3661 |
| **Positive anti-class II immunization** | 0.00 | [-0.08 ; 0.08] | 0.9509 |
| **Biopsy rank** |  |  | 0.0467 |
| **2 (vs. 1)** | -0.06 | [-0.11 ; -0.01] |  |
| **3 (vs. 1)** | -0.10 | [-0.20 ; 0.00] |  |
| **4 (vs. 1)** | 0.11 | [-0.17 ; 0.39] |  |
| **5 (vs. 1)** | -0.10 | [-0.81 ; 0.60] |  |
| **For causes biopsy** | -0.13 | [-0.21 ; -0.06] | 0.0002 |
| **Creatininemia at biopsy (100 μmol/l)** | 0.00 | [-0.10 ; -0.02] | 0.0016 |
| **Post-transplantation time of the biopsy (years)** | 0.00 | [-0.01 ; 0.01] | 0.9940 |
| CI. confidence interval; CMV. cytomegalovirus; Recipient/Donor CMV serology definition. 0: negative donor and recipient; 1: negative donor and positive recipient; 2: positive donor and negative recipient; 3: positive donor and recipient. HLA. human leucocyte antigens. Biopsy group 1: normal/subnormal, group 2: interstitial fibrosis/tubular atrophy (IFTA) grade 2 or 3, group 3: ABMR, group 4: TCMR, group 5: borderline rejection. | | | |

**Supplementary Table 9**. Unadjusted effects of baseline covariates on square root of Tregs in absolute number at the time of biopsy, estimated from linear mixed models including random intercept.

|  | **Mean  difference** | **95% CI** | **p-value** |
| --- | --- | --- | --- |
| **Biopsy group** |  |  | 0.2654 |
| **2 (vs. 1)** | 0.01 | [-0.01 ; 0.03] |  |
| **3 (vs. 1)** | 0.01 | [0.00 ; 0.02] |  |
| **4 (vs. 1)** | 0.01 | [-0.01 ; 0.03] |  |
| **5 (vs. 1)** | 0.01 | [0.00 ; 0.03] |  |
| **Rejection of all types vs. normal/subnormal** | 0.01 | [0.00 ; 0.02] | 0.0528 |
| **Transplantation after 2008** | -0.02 | [-0.04 ; -0.01] | 0.0006 |
| **Recipient age (years)** | 0.00 | [0.00 ; 0.00] | <0.0001 |
| **Male recipient** | 0.01 | [-0.00 ; 0.02] | 0.1230 |
| **Retransplantation** | -0.03 | [-0.04 ; -0.02] | <0.0001 |
| **Renal transplantation** | 0.01 | [0.00 ; 0.02] | 0.3010 |
| **Recurrent initial disease** | 0.01 | [0.00 ; 0.02] | 0.2090 |
| **Delayed graft function** | -0.01 | [-0.02 ; 0.00] | 0.1430 |
| **Cold ischemia time (hours)** | 0.00 | [0.00 ; 0.00] | 0.7810 |
| **History of diabetes** | 0.00 | [-0.01 ; 0.01] | 0.5760 |
| **History of hypertension** | 0.01 | [-0.01 ; 0.02] | 0.4150 |
| **History of cardiovascular disease** | -0.01 | [-0.02 ; 0.00] | 0.0748 |
| **Recipient/Donor CMV serology** |  |  | 0.1407 |
| **1 (vs. 0)** | -0.01 | [-0.02 ; 0.00] |  |
| **2 (vs. 0)** | -0.01 | [-0.02 ; 0.00] |  |
| **3 (vs. 0)** | -0.01 | [-0.02 ; -0.00] |  |
| **Donor age (years)** | 0.00 | [0.00 ; 0.00] | 0.7950 |
| **Male donor** | 0.00 | [-0.01 ; 0.01] | 0.7480 |
| **Deceased donor** | -0.01 | [-0.03 ; 0.00] | 0.0465 |
| **HLA-A-B-DR mismatches > 4** | 0.00 | [-0.02 ; 0.01] | 0.3730 |
| **ABO mismatch** | -0.02 | [-0.05 ; 0.02] | 0.3400 |
| **Depleting induction** | -0.04 | [-0.05 ; -0.03] | <0.0001 |
| **Cyclosporine** | 0.01 | [0.00 ; 0.03] | 0.1220 |
| **Tacrolimus** | -0.01 | [-0.03 ; 0.00] | 0.1150 |
| **Corticosteroids** | -0.03 | [-0.04 ; -0.02] | <0.0001 |
| **Positive anti-class I immunization** | -0.02 | [-0.03 ; -0.01] | 0.0009 |
| **Positive anti-class II immunization** | -0.02 | [-0.03 ; -0.01] | 0.0001 |
| **Positive DSA** | -0.02 | [-0.04 ; -0.01] | 0.0094 |
| **Biopsy rank** |  |  | 0.0815 |
| **2 (vs. 1)** | 0.01 | [0.00 ; 0.02] |  |
| **3 (vs. 1)** | 0.01 | [0.00 ; 0.03] |  |
| **4 (vs. 1)** | 0.02 | [-0.02 ; 0.06] |  |
| **5 (vs. 1)** | 0.01 | [-0.09 ; 0.11] |  |
| **For causes biopsy** | -0.01 | [-0.02 ; 0.00] | 0.0715 |
| **Creatininemia at biopsy (μmol/l)** | 0.00 | [0.00 ; 0.00] | <0.0001 |
| **Post-transplantation time of the biopsy (years)** | 0.00 | [0.00 ; 0.00] | <0.0001 |
| CI. confidence interval; CMV. cytomegalovirus; Recipient/Donor CMV serology definition. 0: negative donor and recipient; 1: negative donor and positive recipient; 2: positive donor and negative recipient; 3: positive donor and recipient. HLA. human leucocyte antigens; DSA. donor-specific antibodies. Biopsy group 1: normal/subnormal, group 2: interstitial fibrosis/tubular atrophy (IFTA) grade 2 or 3, group 3: ABMR, group 4: TCMR, group 5: borderline rejection. | | | |

**Supplementary Table 10**. Results of the multivariable linear mixed model of square root of Tregs in absolute number measured at the time of biopsy (5 biopsy groups).

|  | **Adjusted mean  difference** | **95% CI** | **p-value** |
| --- | --- | --- | --- |
| **Biopsy group** |  |  | 0.2365 |
| **2 (vs. 1)** | 0.00 | [-0.02 ; 0.02] |  |
| **3 (vs. 1)** | 0.01 | [0.00 ; 0.02] |  |
| **4 (vs. 1)** | 0.02 | [0.00 ; 0.04] |  |
| **5 (vs. 1)** | 0.01 | [-0.01 ; 0.02] |  |
| **Transplantation after 2008** | 0.00 | [-0.02 ; 0.03] | 0.8233 |
| **Recipient age (years)** | 0.00 | [0.00 ; 0.00] | 0.0043 |
| **Male recipient** | 0.00 | [0.00 ; 0.01] | 0.2880 |
| **Retransplantation** | 0.00 | [-0.01 ; 0.01] | 0.9095 |
| **Delayed graft function** | 0.00 | [-0.01 ; 0.01] | 0.3619 |
| **Cold ischemia time (hours)** | 0.00 | [0.00 ; 0.00] | 0.0599 |
| **History of cardiovascular disease** | 0.00 | [-0.01 ; 0.01] | 0.7062 |
| **Recipient/Donor CMV serology** |  |  | 0.3912 |
| **1 (vs. 0)** | -0.01 | [-0.02 ; 0.01] |  |
| **2 (vs. 0)** | -0.01 | [-0.02 ; 0.00] |  |
| **3 (vs. 0)** | 0.00 | [-0.01 ; 0.01] |  |
| **Donor age (years)** | 0.00 | [0.00 ; 0.00] | 0.6406 |
| **Male donor** | 0.00 | [0.00 ; 0.01] | 0.3887 |
| **Deceased donor** | -0.01 | [-0.03 ; 0.01] | 0.2260 |
| **HLA-A-B-DR mismatches > 4** | -0.01 | [-0.02 ; 0.00] | 0.1439 |
| **Depleting induction** | -0.05 | [-0.06 ; -0.04] | <0.0001 |
| **Cyclosporine** | 0.02 | [-0.03 ; 0.07] | 0.4305 |
| **Tacrolimus** | 0.03 | [-0.02 ; 0.08] | 0.2162 |
| **Corticosteroids** | -0.02 | [-0.03 ; -0.01] | 0.0021 |
| **Positive anti-class I immunization** | 0.00 | [-0.01 ; 0.01] | 0.8348 |
| **Positive anti-class II immunization** | 0.00 | [-0.01 ; 0.02] | 0.5110 |
| **Positive DSA** | 0.00 | [-0.02 ; 0.02] | 0.8390 |
| **Biopsy rank** |  |  | 0.6875 |
| **2 (vs. 1)** | 0.00 | [0.00 ; 0.01] |  |
| **3 (vs. 1)** | 0.00 | [-0.01 ; 0.02] |  |
| **4 (vs. 1)** | 0.01 | [-0.02 ; 0.05] |  |
| **5 (vs. 1)** | 0.03 | [-0.07 ; 0.12] |  |
| **For causes biopsy** | -0.01 | [-0.02 ; 0.00] | 0.0039 |
| **Creatininemia at biopsy (100 μmol/l)** | -0.01 | [-0.02 ; -0.01] | <0.0001 |
| **Post-transplantation time of the biopsy (years)** | 0.00 | [0.00 ; 0.01] | 0.0029 |
| CI. confidence interval; CMV. cytomegalovirus; Recipient/Donor CMV serology definition. 0: negative donor and recipient; 1: negative donor and positive recipient; 2: positive donor and negative recipient; 3: positive donor and recipient. HLA. human leucocyte antigens; DSA. donor-specific antibodies. Biopsy group 1: normal/subnormal, group 2: interstitial fibrosis/tubular atrophy (IFTA) grade 2 or 3, group 3: ABMR, group 4: TCMR, group 5: borderline rejection. | | | |

**Supplementary Table 11**. Results of the multivariable linear mixed model of square root of Tregs in percentage measured at the time of biopsy (Rejection of all types versus normal/subnormal).

|  | **Adjusted mean  difference** | **95% CI** | **p-value** |
| --- | --- | --- | --- |
| **Rejection of all types vs. normal/subnormal** | 0.06 | [-0.02 ; 0.13] | 0.1038 |
| **Recipient age (years)** | 0.00 | [0.00 ; 0.01] | 0.1897 |
| **Male recipient** | 0.02 | [-0.04 ; 0.09] | 0.4920 |
| **Retransplantation** | -0.08 | [-0.19 ; 0.03] | 0.1579 |
| **Renal transplantation** | -0.15 | [-0.30 ; 0.00] | 0.0522 |
| **Delayed graft function** | -0.05 | [-0.12 ; 0.03] | 0.2155 |
| **Cold ischemia time (hours)** | 0.01 | [0.00 ; 0.01] | 0.0414 |
| **History of diabetes** | 0.00 | [-0.09 ; 0.09] | 0.9939 |
| **Recipient/Donor CMV serology** |  |  | 0.0163 |
| **1 (vs. 0)** | -0.10 | [-0.19 ; -0.02] |  |
| **2 (vs. 0)** | -0.11 | [-0.19 ; -0.02] |  |
| **3 (vs. 0)** | -0.11 | [-0.20 ; -0.03] |  |
| **Donor age (years)** | 0.00 | [-0.01 ; 0.00] | 0.0753 |
| **Male donor** | -0.01 | [-0.08 ; 0.05] | 0.6888 |
| **Deceased donor** | -0.13 | [-0.26 ; 0.00] | 0.0557 |
| **HLA-A-B-DR mismatches > 4** | -0.02 | [-0.09 ; 0.06] | 0.6563 |
| **Depleting induction** | 0.00 | [-0.09 ; 0.09] | 0.9887 |
| **Corticosteroids** | -0.10 | [-0.19 ; -0.00] | 0.0407 |
| **Positive anti-class I immunization** | 0.02 | [-0.05 ; 0.10] | 0.5402 |
| **Positive anti-class II immunization** | -0.01 | [-0.09 ; 0.08] | 0.8930 |
| **Biopsy rank** |  |  | 0.0314 |
| **2 (vs. 1)** | -0.07 | [-0.12 ; -0.02] |  |
| **3 (vs. 1)** | -0.11 | [-0.21 ; -0.01] |  |
| **4 (vs. 1)** | 0.11 | [-0.17 ; 0.38] |  |
| **5 (vs. 1)** | -0.16 | [-0.87 ; 0.55] |  |
| **For causes biopsy** | -0.12 | [-0.19 ; -0.05] | 0.0012 |
| **Creatininemia at biopsy (100 μmol/l)** | -0.07 | [-0.11 ; -0.03] | 0.0007 |
| **Post-transplantation time of the biopsy (years)** | 0.00 | [-0.01 ; 0.01] | 0.9045 |
| CI. confidence interval; CMV. cytomegalovirus; Recipient/Donor CMV serology definition. 0: negative donor and recipient; 1: negative donor and positive recipient; 2: positive donor and negative recipient; 3: positive donor and recipient. HLA. human leucocyte antigens. | | | |

**Supplementary Table 12**. Results of the multivariable linear mixed model of square root of Tregs in absolute number measured at the time of biopsy (Rejection of all types versus normal/subnormal).

|  | **Adjusted mean  difference** | **95% CI** | **p-value** |
| --- | --- | --- | --- |
| **Rejection of all types vs. normal/subnormal** | 0.01 | [0.00 ; 0.02] | 0.0890 |
| **Transplantation after 2008** | 0.00 | [-0.03 ; 0.03] | 0.9518 |
| **Recipient age (years)** | 0.00 | [0.00 ; 0.00] | 0.0038 |
| **Male recipient** | 0.00 | [-0.01 ; 0.01] | 0.4087 |
| **Retransplantation** | 0.00 | [-0.02 ; 0.01] | 0.9134 |
| **Delayed graft function** | -0.01 | [-0.02 ; 0.00] | 0.2359 |
| **Cold ischemia time (hours)** | 0.00 | [0.00 ; 0.00] | 0.0620 |
| **History of cardiovascular disease** | 0.00 | [-0.01 ; 0.01] | 0.5967 |
| **Recipient/Donor CMV serology** |  |  | 0.2031 |
| **1 (vs. 0)** | -0.01 | [-0.02 ; 0.00] |  |
| **2 (vs. 0)** | -0.01 | [-0.02 ; 0.00] |  |
| **3 (vs. 0)** | 0.00 | [-0.01 ; 0.01] |  |
| **Donor age (years)** | 0.00 | [0.00 ; 0.00] | 0.5749 |
| **Male donor** | 0.00 | [-0.01 ; 0.01] | 0.4114 |
| **Deceased donor** | -0.01 | [-0.03 ; 0.01] | 0.2562 |
| **HLA-A-B-DR mismatches > 4** | -0.01 | [-0.02 ; 0.00] | 0.1219 |
| **Depleting induction** | -0.05 | [-0.06 ; -0.04] | <0.0001 |
| **Cyclosporine** | 0.02 | [-0.03 ; 0.07] | 0.4119 |
| **Tacrolimus** | 0.04 | [-0.02 ; 0.09] | 0.1698 |
| **Corticosteroids** | -0.02 | [-0.03 ; -0.01] | 0.0044 |
| **Positive anti-class I immunization** | 0.00 | [-0.01 ; 0.01] | 0.9389 |
| **Positive anti-class II immunization** | 0.00 | [-0.01 ; 0.02] | 0.4876 |
| **Positive DSA** | 0.00 | [-0.02 ; 0.01] | 0.7844 |
| **Biopsy rank** |  |  | 0.8284 |
| **2 (vs. 1)** | 0.00 | [0.00 ; 0.01] |  |
| **3 (vs. 1)** | 0.00 | [-0.01 ; 0.02] |  |
| **4 (vs. 1)** | 0.01 | [-0.03 ; 0.05] |  |
| **5 (vs. 1)** | 0.02 | [-0.08 ; 0.12] |  |
| **For causes biopsy** | -0.01 | [-0.02 ; 0.00] | 0.0176 |
| **Creatininemia at biopsy (100 μmol/l)** | -0.01 | [-0.02 ; -0.01] | <0.0001 |
| **Post-transplantation time of the biopsy (years)** | 0.00 | [0.00 ; 0.01] | 0.0026 |
| CI. confidence interval; CMV. cytomegalovirus; Recipient/Donor CMV serology definition. 0: negative donor and recipient; 1: negative donor and positive recipient; 2: positive donor and negative recipient; 3: positive donor and recipient. HLA. human leucocyte antigens; DSA. donor-specific antibodies. | | | |

**Supplementary Table 13**. Unadjusted effects of baseline covariates on log of the ratio between CD28-CD8+ T cells and Tregs (both in absolute number) measured at the time of biopsy, estimated from linear mixed models including random intercept.

|  | **Mean  difference** | **95% CI** | **p-value** |
| --- | --- | --- | --- |
| **Biopsy group** |  |  | <0.0001 |
| **2 (vs. 1)** | 0.61 | [0.28 ; 0.94] |  |
| **3 (vs. 1)** | 0.57 | [0.34 ; 0.80] |  |
| **4 (vs. 1)** | 0.07 | [-0.33 ; 0.48] |  |
| **5 (vs. 1)** | 0.07 | [-0.25 ; 0.38] |  |
| **Rejection of all types vs. normal/subnormal** | 0.36 | [0.17 ; 0.55] | 0.0002 |
| **Transplantation after 2008** | -0.20 | [-0.50 ; 0.10] | 0.1850 |
| **Recipient age (years)** | 0.01 | [0.00 ; 0.02] | 0.0018 |
| **Male recipient** | -0.09 | [-0.28 ; 0.11] | 0.3720 |
| **Retransplantation** | 0.56 | [0.31 ; 0.80] | <0.0001 |
| **Renal transplantation** | 0.44 | [0.42 ; 1.02] | 0.0069 |
| **Recurrent initial disease** | 0.19 | [-0.03 ; 0.42] | 0.0920 |
| **Delayed graft function** | 0.33 | [0.12 ; 0.53] | 0.0018 |
| **Cold ischemia time (hours)** | 0.03 | [0.01 ; 0.04] | <0.0001 |
| **History of diabetes** | -0.04 | [-0.26 ; 0.18] | 0.7450 |
| **History of hypertension** | -0.08 | [-0.37 ; 0.21] | 0.5930 |
| **History of cardiovascular disease** | 0.09 | [-0.10 ; 0.29] | 0.3570 |
| **Recipient/Donor CMV serology** |  |  | <0.0001 |
| **1 (vs. 0)** | 1.37 | [1.14 ; 1.60] |  |
| **2 (vs. 0)** | 0.71 | [0.48 ; 0.93] |  |
| **3 (vs. 0)** | 1.33 | [1.10 ; 1.56] |  |
| **Donor age (years)** | 0.01 | [0.00 ; 0.02] | 0.0003 |
| **Male donor** | 0.09 | [-0.10 ; 0.28] | 0.3700 |
| **Deceased donor** | 0.42 | [0.13 ; 0.72] | 0.0049 |
| **HLA-A-B-DR mismatches > 4** | -0.17 | [-0.40 ; 0.06] | 0.1400 |
| **ABO mismatch** | -0.43 | [-1.10 ; 0.25] | 0.2160 |
| **Depleting induction** | 0.15 | [-0.04 ; 0.34] | 0.1310 |
| **Cyclosporine** | 0.47 | [0.14 ; 0.81] | 0.0054 |
| **Tacrolimus** | -0.46 | [-0.79 ; -0.13] | 0.0061 |
| **Corticosteroids** | 0.20 | [-0.08 ; 0.48] | 0.1540 |
| **Positive anti-class I immunization** | 0.18 | [-0.02 ; 0.38] | 0.0853 |
| **Positive anti-class II immunization** | 0.25 | [0.04 ; 0.46] | 0.0218 |
| **Positive DSA** | 0.05 | [-0.32 ; 0.41] | 0.8050 |
| **Biopsy rank** |  |  | <0.0001 |
| **2 (vs. 1)** | 0.53 | [0.42 ; 0.65] |  |
| **3 (vs. 1)** | 0.81 | [0.57 ; 1.04] |  |
| **4 (vs. 1)** | 1.00 | [0.33 ; 1.67] |  |
| **5 (vs. 1)** | 1.15 | [-0.50 ; 2.80] |  |
| **For causes biopsy** | 0.35 | [0.18 ; 0.51] | <0.0001 |
| **Creatininemia at biopsy (μmol/l)** | 0.00 | [0.00 ; 0.00] | 0.0045 |
| **Post-transplantation time of the biopsy (years)** | 0.05 | [0.03 ; 0.07] | <0.0001 |
| CI. confidence interval; CMV. cytomegalovirus; Recipient/Donor CMV serology definition. 0: negative donor and recipient; 1: negative donor and positive recipient; 2: positive donor and negative recipient; 3: positive donor and recipient. HLA. human leucocyte antigens; DSA. donor-specific antibodies. Biopsy group 1: normal/subnormal, group 2: interstitial fibrosis/tubular atrophy (IFTA) grade 2 or 3, group 3: ABMR, group 4: TCMR, group 5: borderline rejection. | | | |
